# LC–MS/MS Chemical Profiling and HPLC/PDA Quantification of *Hypericum perforatum* Extract and Its Anti-Inflammatory Properties

**DOI:** 10.3390/ph19081136

**Published:** 2026-07-23

**Authors:** Sang-Yun Lee, Yu Ri Jeong, Hak-Dong Lee, Hyoung Su Park, Hye-Jeong See, Ah Young Lee, Sanghyun Lee

**Affiliations:** 1 Department of Plant Science and Technology, Chung-Ang University, Anseong 17546, Republic of Korea; andylsy@naver.com (S.-Y.L.); gkrehd1234@naver.com (H.-D.L.); 2 Department of Food and Nutrition, Gyeongsang National University, Jinju 52828, Republic of Korea; wjddbfl5675@gmail.com; 3 Natural Product Institute of Science and Technology, Anseong 17546, Republic of Korea; 4 Nutrition R&D, Maeil Dairies Co., Ltd., Pyeongtaek 17714, Republic of Korea; parkhs@maeil.com (H.S.P.); hyejeong.see@maeil.com (H.-J.S.)

**Keywords:** *Hypericum perforatum*, quercetin glycosides, LC–MS/MS, HPLC/PDA, nitric oxide, RAW264.7 macrophages, anti-inflammatory activity

## Abstract

**Background/Objectives:** *Hypericum perforatum* L. is a medicinal plant containing diverse specialized metabolites, including flavonoids, naphthodianthrones, phloroglucinols, and phenolic acids. However, the relationship between its quantitative flavonoid composition and extract-level anti-inflammatory activity remains insufficiently characterized. This study aimed to profile the chemical constituents of *H. perforatum* extract (HPE), quantify selected flavonoid markers, and relate these data to nitric oxide (NO) inhibitory activity in LPS-stimulated RAW 264.7 macrophages. **Methods:** HPE was chemically characterized by LC–MS/MS, and selected flavonoid constituents were quantified using HPLC/PDA analysis. Cell viability and NO production were evaluated in RAW 264.7 macrophages using MTT and Griess assays, respectively. **Results:** LC–MS/MS profiling tentatively identified 36 compounds in HPE, with flavonoid-related metabolites representing the largest portion of the annotation list. Multiple quercetin-derived glycosides and conjugates were detected, indicating a flavonoid-rich phenolic profile. HPLC/PDA analysis showed that hyperoside (**2**) was the most abundant quantified marker at 12.44 mg/g extract, followed by rutin (**1**, 9.18 mg/g), quercetin (**4**, 6.64 mg/g), and avicularin (**3**, 5.55 mg/g), whereas amentoflavone (**5**) was detected only at trace level. At non-cytotoxic concentrations, HPE significantly suppressed LPS-induced NO production, iNOS protein expression, and the mRNA expression of the pro-inflammatory cytokines Tnf and Il6 in RAW 264.7 macrophages. Compounds **1**–**4** also reduced NO production and iNOS protein expression, although their effects on cytokine mRNA expression differed among compounds. **Conclusions:** HPE exhibited anti-inflammatory potential in LPS-stimulated macrophages, as shown by inhibition of NO production, downregulation of iNOS protein expression, and reduced Tnf/Il6 mRNA expression. However, extract-equivalent comparison indicated that the extract-level response could not be directly assigned to any single quantified flavonoid marker. The broader flavonoid-rich phenolic matrix may provide a plausible context for this response, but additive or synergistic interactions among constituents were not directly demonstrated and require further validation.

## 1. Introduction

Inflammation is a protective biological response that becomes pathological when dysregulated, thereby contributing to the onset and progression of numerous chronic diseases [1]. At the molecular level, inflammatory processes involve the activation of signaling pathways that regulate the production of inflammatory mediators, including pro-inflammatory cytokines, reactive oxygen species (ROS), and nitric oxide (NO) [2]. Among these mediators, NO generated by inducible nitric oxide synthase (iNOS) in activated macrophages plays a central role in amplifying inflammatory responses and promoting tissue damage when produced in excess [3]. Therefore, suppression of excessive NO production is considered an important strategy for the control of inflammatory responses.

Because inflammation is regulated through multiple interconnected signaling pathways, considerable research has focused on identifying bioactive compounds capable of modulating multiple molecular targets simultaneously [4]. In this context, plant-derived compounds have attracted substantial attention as potential anti-inflammatory agents because of their structural diversity, pleiotropic biological activities, and generally favorable safety profiles [5]. Accordingly, in vitro assays measuring NO production in lipopolysaccharide (LPS)-stimulated macrophages have been widely employed as a primary screening platform for evaluating the anti-inflammatory potential of natural products [6,7,8].

*Hypericum perforatum* L. (St. John’s wort), a member of the family Hypericaceae, is a perennial herbaceous species widely distributed throughout temperate regions. The plant is morphologically characterized by opposite leaves containing translucent glandular structures and yellow flowers with prominent stamens [9]. Although *H. perforatum* is most widely recognized for its use in the treatment of depressive disorders, previous studies have also reported a broad spectrum of biological activities, including antioxidant and anti-inflammatory effects [10]. Due to these pharmacological properties, *H. perforatum* continues to attract interest as a promising source of bioactive natural products [11].

Phytochemical studies of *H. perforatum* have extensively documented several characteristic constituents, including hyperforin and hypericin derivatives, which are commonly used for quality control and pharmacological evaluation [12]. In addition to these well-established markers, *H. perforatum* contains a diverse range of phenolic and flavonoid-related compounds, including flavonol glycosides and biflavonoids [13]. Among them, quercetin-type flavonoids such as rutin, hyperoside, and related glycosides are frequently detected constituents of *H. perforatum* extracts and are often used to support interpretations of their phenolic-rich chemical profiles. Structurally, these flavonoid derivatives differ in glycosylation pattern, aglycone form, and degree of molecular association, and such differences may influence their physicochemical properties, cellular availability, and interactions with molecular targets involved in inflammatory signaling [14,15,16,17].

Although the phytochemical composition and biological activities of *H. perforatum* have been widely investigated, the interpretation of extract-level activity remains challenging because plant extracts contain multiple constituents that may contribute differently to the overall biological response [18,19]. In many phytochemical studies, the presence of flavonoid-rich profiles is used to support anti-inflammatory interpretations; however, the actual concentrations of individual marker compounds within a biologically active extract dose are not always compared with the concentrations required for activity of the corresponding isolated compounds under the same assay conditions. This comparison is particularly important in chemically complex extracts, because a compound may exhibit activity in isolated form while being present in the extract at a concentration too low to account for the extract-level response by itself [20].

Accordingly, the present study aimed to characterize the chemical composition of *H. perforatum* extract (HPE) and to provide a quantitative context for evaluating whether selected flavonoid markers could account for the extract-level NO inhibitory activity. HPE was initially profiled using untargeted liquid chromatography–tandem mass spectrometry (LC–MS/MS) to obtain an overview of its phenolic and flavonoid-related constituents, followed by targeted high-performance liquid chromatography/photodiode array detector (HPLC/PDA) analysis to quantify selected flavonoid markers representing the flavonoid-rich fraction of the extract. The inhibitory effects of HPE and the quantified constituents on NO production were subsequently evaluated in LPS-stimulated macrophages in parallel with cell viability assays. In addition, the effects of HPE on inflammatory mediator expression were investigated by analyzing iNOS protein expression and the mRNA expression of TNF-α and IL-6 to determine whether the observed NO inhibitory activity was accompanied by modulation of inflammatory responses at the molecular level.

## 2. Results and Discussion

### 2.1. LC–MS/MS Analysis

LC–MS/MS profiling was performed to characterize the overall phytochemical composition of HPE. Raw data were processed using a library-based untargeted workflow, and candidate annotations were generated by matching accurate mass values and MS/MS spectral data with entries in spectral libraries (Figures S1 and S2). Annotation confidence was classified according to the Metabolomics Standards Initiative (MSI) framework, and the assigned confidence levels are indicated by superscript numbers in Table 1 and Table 2. MSI Level 1 was assigned when the precursor accurate mass, retention time, and MS/MS spectrum matched an in-house library entry generated from an authentic standard under identical LC–MS/MS conditions. MSI Level 2 was assigned when the precursor accurate mass and MS/MS spectrum matched reference-library data, but confirmation using an authentic standard under identical analytical conditions was not available. MSI Level 3 was assigned when the reported compound name represented the best-matching database or library candidate, but the exact positional, glycosidic, or stereochemical isomer could not be unambiguously determined. This terminology is consistent with commonly accepted metabolomics reporting guidelines, in which definitive compound identification generally requires direct comparison with authentic standards.

**Figure 1:**
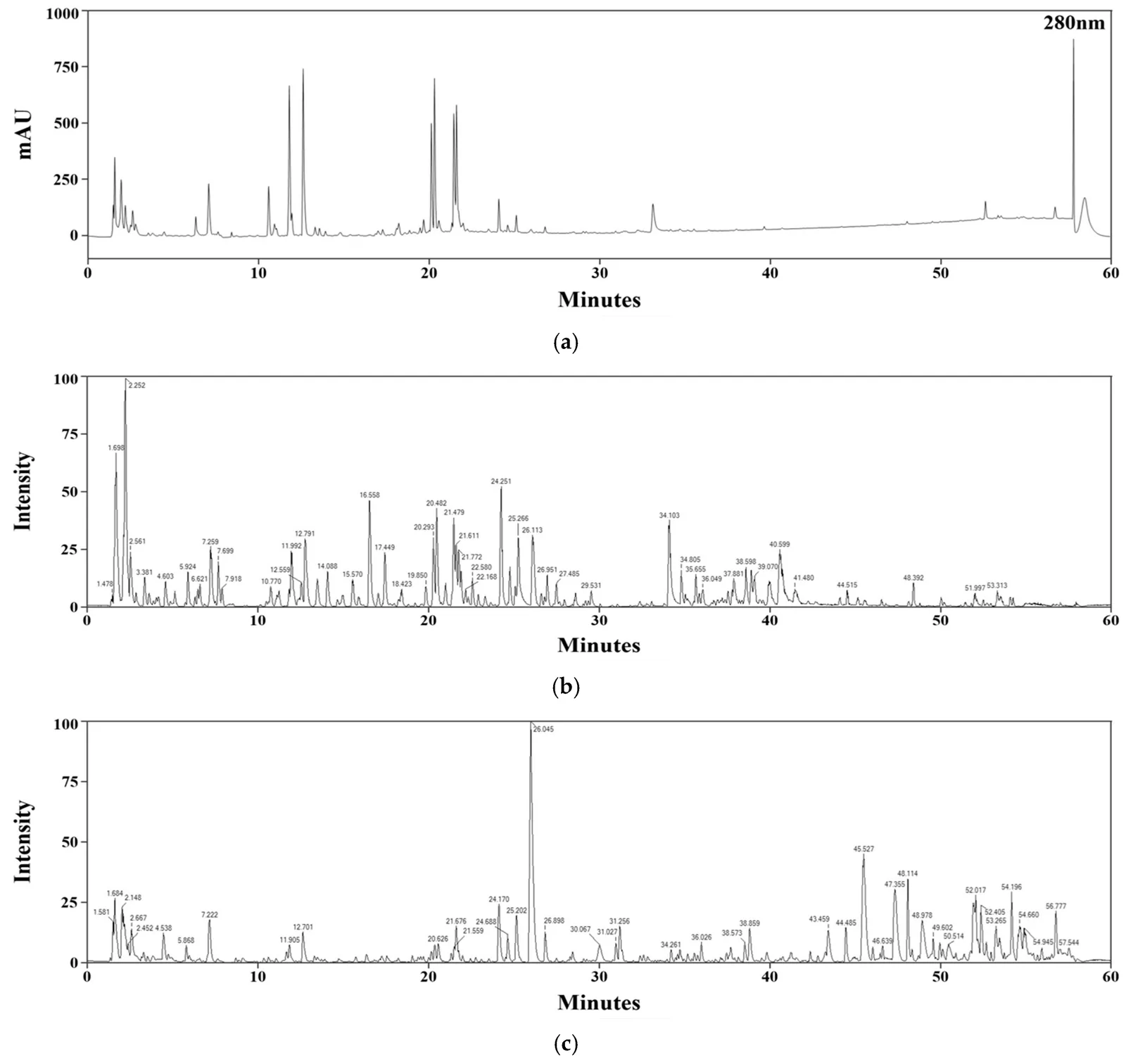
UV chromatogram of HPE at 280 nm (**a**) and base peak chromatograms of HPE acquired in negative ion mode (**b**) and positive ion mode (**c**).

A total of 36 compounds were tentatively identified in HPE (Figure 1, Table 1 and Table 2). These compounds were mainly assigned to phenolic acid derivatives, hydroxycinnamoylquinic acid derivatives, flavan-3-ols, procyanidins, xanthones, flavonol/flavone glycosides, flavonoid aglycones, and biflavonoids. Among these categories, flavonoid-related metabolites represented the most frequently annotated class. Specifically, 22 of the 36 tentatively identified compounds were classified as flavonoid-related metabolites, corresponding to approximately 61% of the annotation list on a compound-number basis. Therefore, the present HPE sample can be described as possessing a flavonoid-rich phenolic profile at the annotation level. However, this proportion should not be interpreted as a direct reflection of concentration or chromatographic peak-area distribution.

The flavonoid profile was dominated primarily by flavonol derivatives. In particular, several quercetin-derived glycosides and conjugates were detected, including rutin, hyperoside, quercetin 3-*O*-glucuronide, avicularin, spiraeoside, quercitrin, and quercetin-3-*O*-glucose-6″-acetate. These compounds share a common quercetin-type flavonol scaffold but differ in their attached sugar moieties or conjugating groups. Their repeated detection indicates that quercetin-type flavonols occur in HPE not only as free aglycones but also as structurally diverse conjugated derivatives. This pattern is consistent with previous phytochemical reports on *H. perforatum*, in which quercetin glycosides such as rutin, hyperoside, avicularin, quercitrin, and related derivatives have been recognized as characteristic polyphenolic constituents [21].

The coexistence of flavonol aglycones and glycosylated derivatives is also chemically significant. In plants, glycosylation represents one of the major modification pathways of phenylpropanoid- and flavonoid-derived metabolites and can influence solubility, stability, toxicity, intracellular compartmentalization, and biological activity [22]. Therefore, the presence of multiple quercetin-type glycosides in HPE should not be regarded merely as repeated detection of structurally similar compounds, but rather as evidence of structural diversification originating from a common flavonol scaffold [23].

In addition to quercetin-related glycosides, HPE also contained flavonoid aglycones such as quercetin, luteolin, and kaempferol, as well as amentoflavone, a biflavonoid previously reported in *Hypericum* species [24].

Besides flavonoids, HPE also contained phenolic acids and hydroxycinnamoylquinic acid derivatives, including protocatechuic acid, protocatechuic aldehyde, neochlorogenic acid, chlorogenic acid, cryptochlorogenic acid, and several coumaroylquinic acid isomers. The presence of these compounds indicates that the extract also contains upstream phenylpropanoid-derived metabolites. Nevertheless, the most prominent feature of the LC–MS/MS annotation profile was the high representation of flavonoid-related compounds, particularly quercetin-type flavonol derivatives. Comparable phytochemical profiles have also been reported for Syrian and Turkish *H. perforatum* extracts, in which several quercetin-derived constituents were likewise detected [12,21]. This qualitative agreement supports the consistency of the present HPE profile with the reported phytochemical characteristics of *H. perforatum*, although the relative distribution of individual constituents may vary with plant origin and extraction conditions.

In summary, LC–MS/MS profiling demonstrated that HPE possesses a flavonoid-centered phenolic composition characterized by multiple quercetin-derived glycosides and conjugates, together with flavonoid aglycones and a biflavonoid constituent. This phytochemical profile provided the basis for the subsequent HPLC/PDA analysis, which was conducted to obtain standard-based quantitative information on selected flavonoid constituents present in the same extract.

### 2.2. HPLC/PDA Analysis

**Figure 2:**
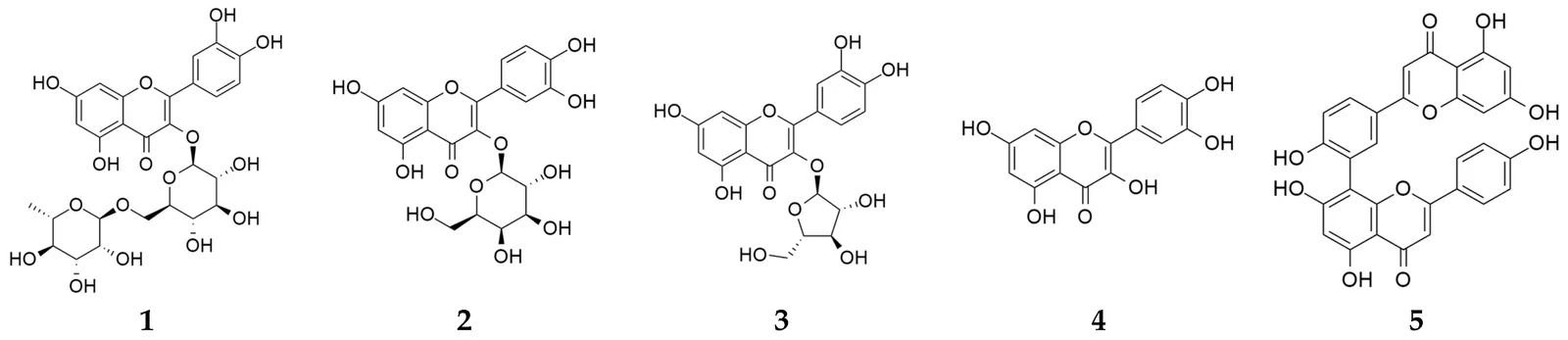
Chemical structures of compounds **1**–**5**. Compounds: rutin (**1**), hyperoside (**2**), avicularin (**3**), quercetin (**4**), and amentoflavone (**5**).

**Figure 3:**
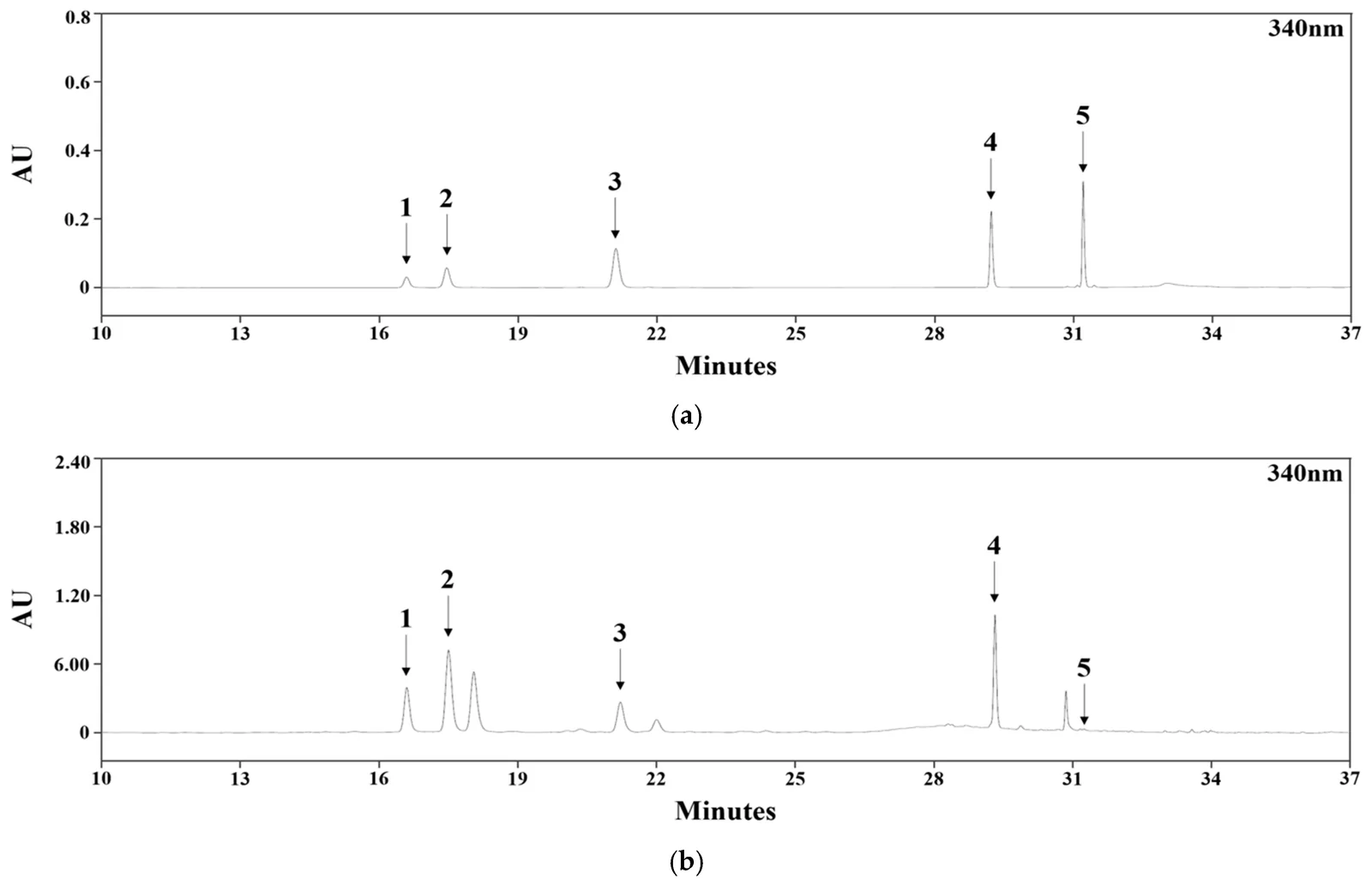
HPLC/PDA chromatograms of compounds **1**–**5** (**a**) and HPE (**b**). Compounds: rutin (**1**), hyperoside (**2**), avicularin (**3**), quercetin (**4**), and amentoflavone (**5**).

HPLC/PDA analysis was conducted to obtain standard-based quantitative information on selected flavonoid constituents in HPE. Based on the flavonoid-rich LC–MS/MS profile and previously reported flavonoid constituents of *H. perforatum*, rutin (**1**), hyperoside (**2**), avicularin (**3**), quercetin (**4**), and amentoflavone (**5**) were selected as target compounds. Compounds **1**–**4** were selected because they corresponded to clearly resolved flavonoid peaks in the HPLC/PDA chromatogram, whereas amentoflavone (**5**) was included as a representative biflavonoid constituent reported in *Hypericum* species. These compounds also provided structural coverage within the flavonoid-rich fraction, including quercetin glycosides with different sugar residues, the corresponding aglycone, and a biflavonoid constituent. In addition, the same compounds were evaluated in the subsequent NO assay, allowing the quantitative data to be directly compared with compound-level biological effects. Thus, this marker panel was used to support quantitative interpretation of the flavonoid-rich fraction of HPE, rather than to provide comprehensive standardization of all characteristic constituents of the extract. The chemical structures of these reference compounds are shown in Figure 2. Chromatograms of the reference standards and HPE are presented in Figure 3. Peaks corresponding to compounds **1**–**5** were assigned by comparison with the retention times of authentic standards, and their identities were further supported by co-elution in spike tests performed with the corresponding reference compounds. Calibration curves were generated over the concentration range of 15.625–500 μg/mL, and compounds **1**–**4** exhibited excellent linearity, with *R*^2^ values of 0.9995, 0.9998, 0.9999, and 0.9997, respectively (Table 3).

Among the quantified compounds, hyperoside (**2**) was the most abundant quantified marker at 12.44 mg/g extract, followed by rutin (**1**) at 9.18 mg/g extract, quercetin (**4**) at 6.64 mg/g extract, and avicularin (**3**) at 5.55 mg/g extract (Table 3). Amentoflavone (**5**) was detected only at trace levels under the present HPLC/PDA conditions. The total content of the four quantifiable flavonoids was 33.81 mg/g extract, corresponding to approximately 3.38% of the extract weight. Within this quantified marker set, rutin (**1**) and hyperoside (**2**) together accounted for 21.62 mg/g extract, representing approximately 64% of the quantified flavonoid markers. These results indicate that the quantified flavonoid profile of HPE was dominated by quercetin glycosides rather than by the corresponding free aglycone. Direct comparison with published quantitative data further illustrates compositional variability among HPE. In a previous methanolic extract, rutin (**1**), hyperoside (**2**), and quercetin (**4**) were reported at 1.124, 0.805, and 0.039 mg/g, respectively, whereas all three were present at higher levels in the present 80% EtOH-derived HPE, with hyperoside (**2**) rather than rutin (**1**) as the predominant marker [12]. These differences may reflect variation in plant origin, extraction procedure, and analytical conditions, highlighting the importance of interpreting the biological findings in relation to the chemically characterized extract evaluated here.

This quantitative distribution was consistent with the LC–MS/MS profile, in which multiple quercetin-derived glycosides and conjugates were detected. Compounds **1**–**3** are all quercetin glycosides that differ in their attached sugar residues, whereas quercetin (**4**) represents the corresponding aglycone form. This distribution is consistent with a quercetin-type flavonol pool in HPE that is represented mainly by conjugated glycosidic forms rather than as free aglycones. In plant tissues, flavonoid glycosylation is closely associated with metabolic homeostasis because it reduces the chemical reactivity of phenolic aglycones, enhances compatibility with aqueous intracellular environments, and facilitates sequestration into vacuoles or other storage compartments [25,26,27]. Therefore, the predominance of rutin (**1**) and hyperoside (**2**) over quercetin (**4**) may reflect the physiological tendency of *H. perforatum* to accumulate quercetin-derived metabolites primarily as stable glycosidic reserves rather than as free aglycone forms. Overall, the quantitative predominance of rutin (**1**) and hyperoside (**2**) supports the interpretation that the present HPE sample possesses a glycoside-rich flavonol profile rather than one dominated by aglycone-type flavonoids.

The lower but measurable content of quercetin (**4**) is also noteworthy. Quercetin (**4**) was present at 6.64 ± 0.08 mg/g extract, corresponding to approximately 20% of the four quantified flavonoid markers. From the perspective of plant metabolism, quercetin (**4**) can be regarded as a core aglycone scaffold from which multiple quercetin glycosides are biosynthesized through glycosylation reactions [28]. Therefore, its coexistence with compounds **1–3** suggests that the quantified flavonol composition of HPE reflects a quercetin-centered metabolic pool consisting of both the free aglycone scaffold and its downstream glycosylated derivatives. Although glycosides were quantitatively predominant, the measurable amount of quercetin (**4**) indicates that the extract contains not only accumulated glycosidic end products but also retains the corresponding aglycone within the flavonol metabolic network. In contrast, amentoflavone (**5**) was detected only at trace levels despite its presence in the LC–MS/MS profile. This observation suggests that although amentoflavone (**5**) is a characteristic biflavonoid reported in *Hypericum* species, it is unlikely to represent a quantitatively dominant marker in the present HPE sample [29].

Collectively, the HPLC/PDA results demonstrated that HPE contains measurable levels of selected quercetin-type flavonoids. Hyperoside (**2**) was quantified as the most abundant marker, followed by rutin (**1**), quercetin (**4**), and avicularin (**3**), whereas amentoflavone (**5**) was present only at trace levels. These findings provide a quantitative basis for describing the present HPE sample as having a quercetin–glycoside-rich profile within the selected flavonoid marker panel. Although this targeted panel does not represent all constituents detected by LC–MS/MS, it captures the major standard-confirmed flavonoid markers relevant to the subsequent compound-level NO assay. In the context of the subsequent biological assays, this targeted quantitative information is particularly valuable because it enables interpretation of NO inhibitory activity in relation to actual marker concentrations rather than relying solely on qualitative LC–MS/MS annotations.

### 2.3. Cytotoxicity of HPE and Its Compounds ***1***–***5*** on RAW 264.7 Macrophages

**Figure 4:**
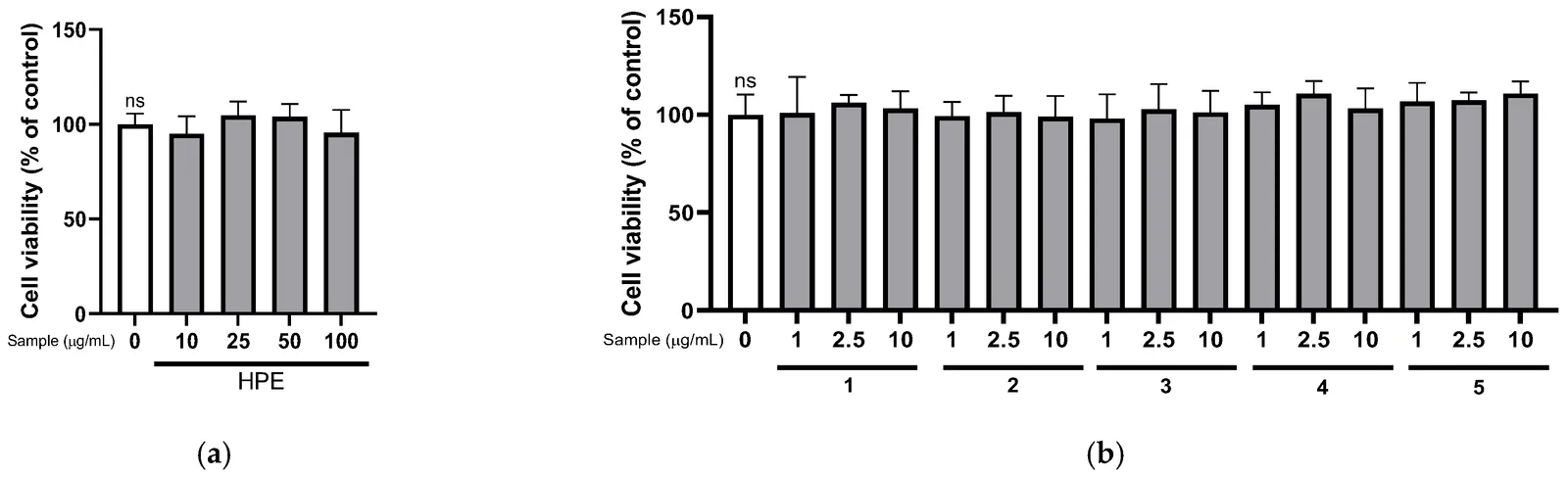
Cytotoxicity of HPE and compounds **1**–**5** on RAW 264.7 macrophages. Cell viability of macrophages treated with HPE (10–100 μg/mL) (**a**). Cell viability of macrophages treated with compounds **1**–**5** at the indicated concentrations (1–10 μg/mL) (**b**). Cell viability is expressed as a percentage of the untreated control. Data are presented as mean ± SD (*n* = 6). ns, not significant; *p* < 0.05 was considered statistically significant. Compounds: rutin (**1**), hyperoside (**2**), avicularin (**3**), quercetin (**4**), and amentoflavone (**5**).

RAW 264.7 cells are widely used as an in vitro model for studies of immune function and inflammation [30,31]. To evaluate the potential cytotoxicity of HPE and compounds **1**–**5**, a 3-(4,5-dimethylthiazol-2-yl)-2,5-diphenyltetrazolium bromide (MTT) assay was performed using RAW 264.7 macrophages. Treatment with HPE at concentrations up to 100 μg/mL did not produce any statistically significant reduction in cell viability compared with the untreated control group (Figure 4a). Similarly, compounds **1**–**5** showed no detectable cytotoxicity at concentrations ranging from 1 to 10 μg/mL (Figure 4b).

These findings indicate that the concentration ranges used in the subsequent NO inhibition assay were not associated with measurable cytotoxic effects. Therefore, changes in NO production observed under these experimental conditions can be interpreted as functional modulation of inflammatory responses rather than secondary effects resulting from reduced cell viability.

### 2.4. Inhibitory Effect of HPE and Its Compounds ***1***–***5*** on NO Production in LPS-Stimulated RAW 264.7 Macrophages

**Figure 5:**
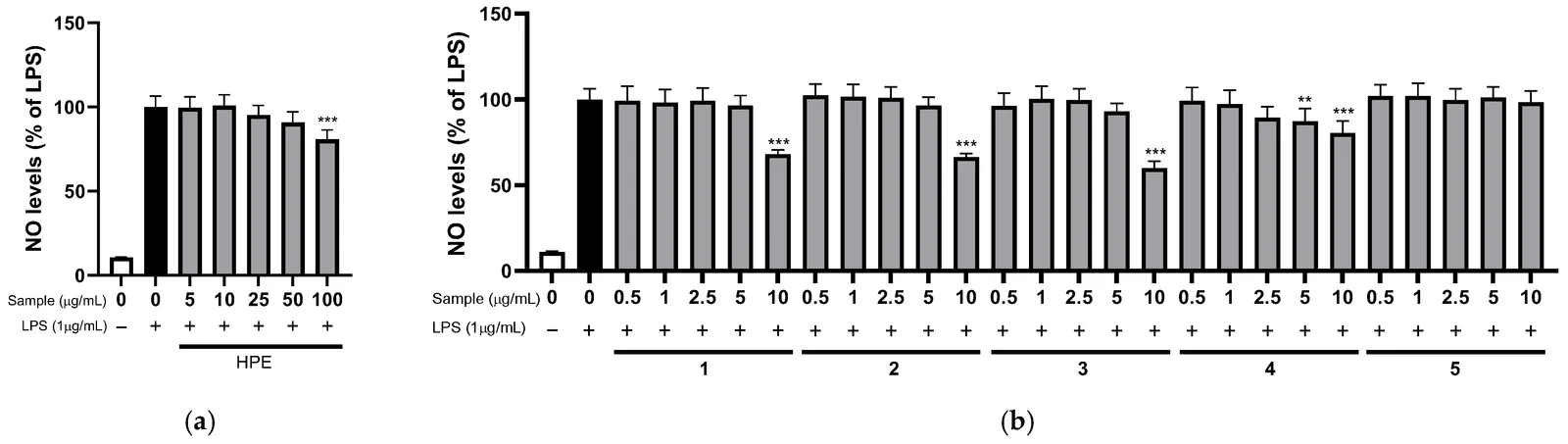
Effects of HPE and compounds **1**–**5** on NO production in LPS-stimulated macrophages. HPE (5–100 μg/mL) (**a**) and compounds **1**–**5** (0.5–10 μg/mL) (**b**) were evaluated for their inhibitory effects on NO production in the presence of LPS (1 μg/mL). NO levels are expressed as a percentage of the LPS-treated group. Bar graph representations: white bar, untreated control; black bar, LPS-induced control; grey bar, sample-treated groups in the presence of LPS. Data are presented as mean ± SD (*n* = 6). Statistical significance was defined as ** *p* < 0.01 and *** *p* < 0.001 compared with the LPS-treated group. Compounds: rutin (**1**), hyperoside (**2**), avicularin (**3**), quercetin (**4**), and amentoflavone (**5**).

LPS stimulation in RAW 264.7 macrophages induces inflammatory responses through activation of intracellular signaling pathways, resulting in upregulation of iNOS and subsequent NO production [32]. Although NO plays an important role in host defense and immune regulation, excessive NO production by activated macrophages can contribute to oxidative stress and inflammatory tissue damage [31,32,33]. Accordingly, inhibition of excessive NO production in LPS-stimulated macrophages is widely used as an indicator of anti-inflammatory potential in natural product research [34,35]. In the present study, HPE significantly suppressed LPS-induced NO production at 100 μg/mL, whereas lower concentrations exhibited only limited inhibitory effects (Figure 5a). Following the extract-level evaluation, the inhibitory effects of compounds **1**–**5** on NO production were examined individually to further assess their possible relevance to the observed response.

The inhibitory effects of compounds **1**–**5** on NO production were subsequently evaluated to determine whether the quantified flavonoid markers also exhibited activity at the individual compound level. Among the tested compounds, rutin (**1**), hyperoside (**2**), and avicularin (**3**) significantly inhibited NO production at 10 μg/mL, whereas quercetin (**4**) significantly reduced NO production at both 5 and 10 μg/mL (Figure 5b). In contrast, amentoflavone (**5**) did not significantly inhibit NO production within the tested concentration range. This differs from a previous report showing that amentoflavone (**5**) inhibited NF-κB activation and iNOS induction in macrophages, which may reflect differences in treatment concentration or assay conditions [36]. Given its trace-level occurrence in HPE, the present data do not support a major quantitative contribution of amentoflavone (**5**) to the extract-level response. These results indicate that the major quantified quercetin-type flavonoids in HPE, particularly compounds **1**–**4**, are capable of suppressing LPS-induced NO production without causing detectable cytotoxicity.

These compound-level findings are broadly consistent with previous cell-based studies and provide a basis for considering upstream signaling mechanisms not examined in the present study. Rutin (**1**) has been reported to reduce LPS-induced NO, PGE2, TNF-α, and IL-6 production in RAW 264.7 macrophages [37]. Hyperoside (**2**) was shown to suppress NO production, iNOS expression, and pro-inflammatory cytokine production through modulation of p38 and NF-κB signaling in LPS-stimulated microglial cells [38], whereas avicularin (**3**) inhibited NO production and iNOS expression in RAW 264.7 macrophages through suppression of ERK signaling [39]. Quercetin (**4**) has likewise been reported to attenuate the iNOS/NO system and pro-inflammatory gene expression [40,41,42]. Collectively, these findings suggest that regulation of NF-κB- and MAPK-associated signaling may provide an upstream mechanistic context for the reductions in NO production, iNOS expression, and inflammatory cytokine expression observed in the present study. However, because these signaling pathways were not directly examined here, their involvement remains to be verified.

Importantly, however, the contribution of these compounds to the extract-level activity should be interpreted with caution. Based on the HPLC/PDA-derived quantitative data, HPE at 100 μg/mL was estimated to contain approximately 1.24 μg/mL hyperoside (**2**), 0.92 μg/mL rutin (**1**), 0.66 μg/mL quercetin (**4**), and 0.56 μg/mL avicularin (**3**). These estimated concentrations are lower than the concentrations at which the isolated compounds exhibited statistically significant NO inhibitory activity in the present assay system. The total anti-inflammatory activity of HPE is not merely the mathematical sum of rutin (**1**), hyperoside (**2**), avicularin (**3**), and quercetin (**4**). Instead, the anti-inflammatory efficacy of HPE is more appropriately interpreted as a multi-target synergistic network driven by the extract’s complex phytochemical matrix. Accumulating literature supports this phenomenon, demonstrating that specific combinations of plant-derived phytochemicals can markedly enhance anti-inflammatory and antioxidant efficacy compared to single isolated compounds. For instance, the co-treatment of rutin and quercetin has been shown to exert synergistic radical scavenging activity rather than the effect of single compound [43,44,45]. In our findings, while an individual flavonoid like rutin or quercetin within the extract is insufficient to trigger a biological response, their co-existence-alongside the other 32 identified phenolics- cooperatively triggers anti-inflammatory signaling pathways.

Collectively, these findings indicate that HPE contains quantified quercetin-type flavonoid markers with compound-level NO inhibitory potential. However, because their estimated extract-equivalent concentrations were below the individually active concentrations observed in the present assay, the extract-level NO inhibitory activity cannot be directly assigned to any single quantified marker. Thus, the flavonoid-rich matrix of HPE provides a chemically plausible context for the observed activity, but the specific contribution of individual or combined constituents remains to be demonstrated in future studies.

### 2.5. Effect of HPE and Its Compounds ***1***–***4*** on iNOS Protein Expression in LPS-Stimulated RAW 264.7 Macrophages

**Figure 6:**
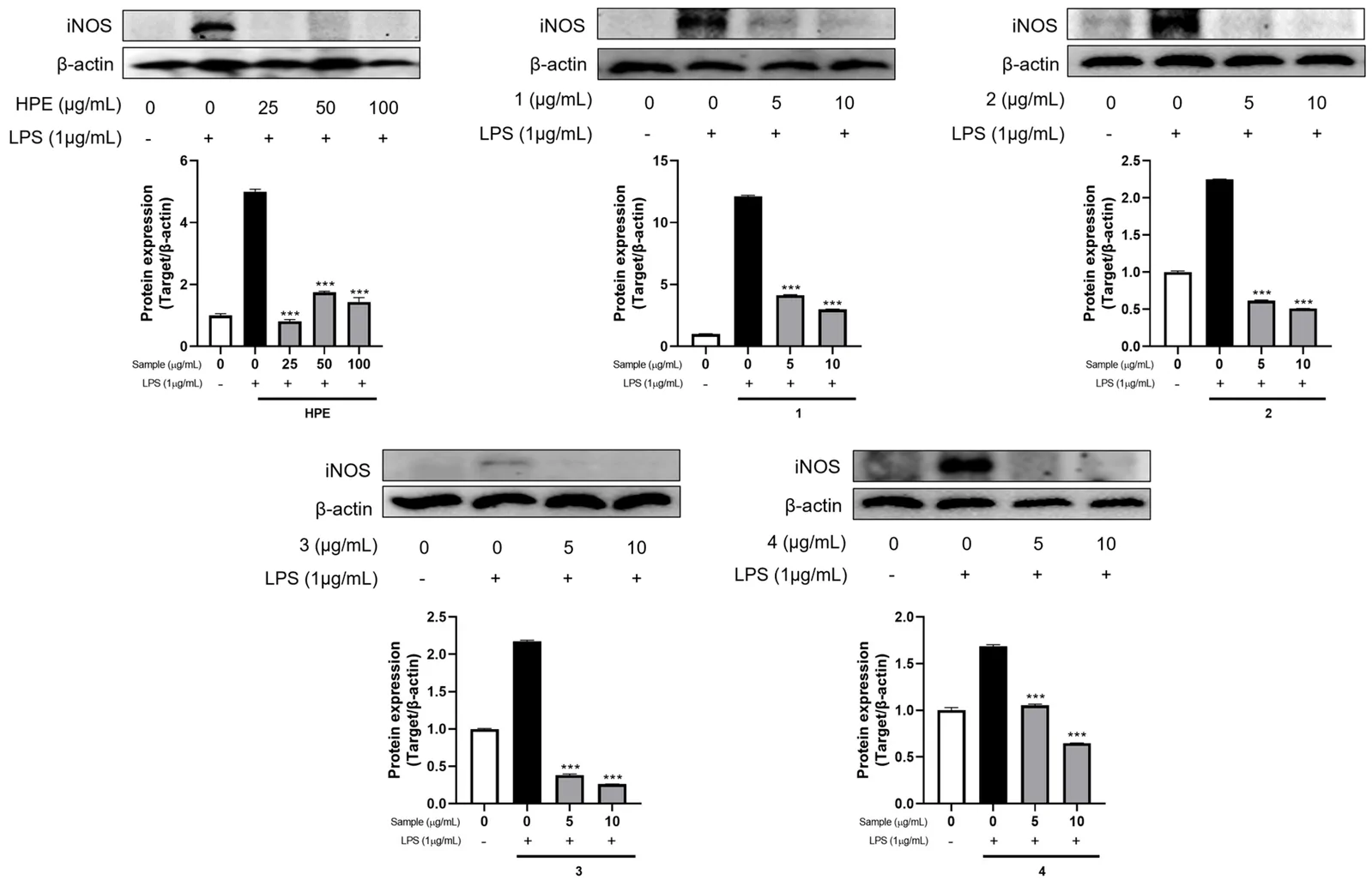
Effects of HPE and compounds **1**–**4** on iNOS protein expression in LPS-stimulated RAW 264.7 macrophages. HPE (25–100 μg/mL) and compounds **1**–**4** (5 and 10 μg/mL) were evaluated for their effects on iNOS protein expression following stimulation with LPS (1 μg/mL). iNOS protein expression was normalized to β-actin and is presented as a percentage of the untreated control group. Bar graph representations: white bar, untreated control; black bar, LPS-induced control; grey bar, sample-treated groups in the presence of LPS. Data are expressed as the mean ± SD. Statistical significance was defined as *** *p* < 0.001 compared with the LPS-treated group. Compounds: rutin (**1**), hyperoside (**2**), avicularin (**3**), and quercetin (**4**).

iNOS is an inducible enzyme that is highly expressed in activated macrophages following LPS stimulation and is responsible for the sustained production of large amounts of NO [46]. While physiological concentrations of NO play crucial roles in host defense and intracellular signaling, excessive NO production contributes to inflammatory tissue injury by reacting with superoxide anions to generate highly reactive nitrogen species, including peroxynitrite [47,48]. Therefore, targeting iNOS expression represents a critical therapeutic strategy for modulating macrophage-mediated chronic inflammatory disorders. To further investigate whether the observed reduction in NO production was associated with the regulation of inflammatory protein expression at the molecular level, the protein expression of iNOS, a key enzyme responsible for NO synthesis during inflammatory responses, was evaluated by Western blot analysis (Figure 6 and Figure S3).

Our findings indicated that LPS stimulation markedly upregulated iNOS protein expression compared with the untreated control group. In contrast, pretreatment with HPE effectively and significantly downregulated LPS-induced iNOS protein expression across all tested concentrations (25–100 μg/mL). Furthermore, compounds **1**–**4** significantly attenuated iNOS protein expression at both 5 and 10 μg/mL, suggesting that the reduction in NO production by HPE and selected flavonoid markers is associated, at least in part, with downregulation of iNOS expression. LPS-induced iNOS expression is primarily regulated through the activation of TLR4/NF-κB signaling pathway, as well as mitogen-activated protein kinase (MAPK) signaling cascades [49,50,51]. Although additional mechanistic experiments tracking the phosphorylation and nuclear translocation of signaling molecules such as NF-κB subunits or MAPKs are required, our current findings demonstrate that HPE and its flavonoids exert their anti-inflammatory properties by suppression of iNOS, one of the most critical enzymes of downstream inflammatory cascades.

### 2.6. Effect of HPE and Its Compounds ***1***–***4*** on Pro-Inflammatory Cytokine Gene in LPS-Stimulated RAW 264.7 Macrophages

TNF-α and IL-6 are key pro-inflammatory cytokines that orchestrate both the initiation and progression of inflammatory responses [52,53]. Upon LPS stimulation, activated macrophages rapidly release TNF-α, which subsequently promotes the production of additional inflammatory mediators, including IL-6, thereby amplifying the inflammatory cascade [54]. Persistent overexpression of these cytokines has been implicated in the development of chronic inflammatory disorders and tissue injury [55].

**Figure 7:**
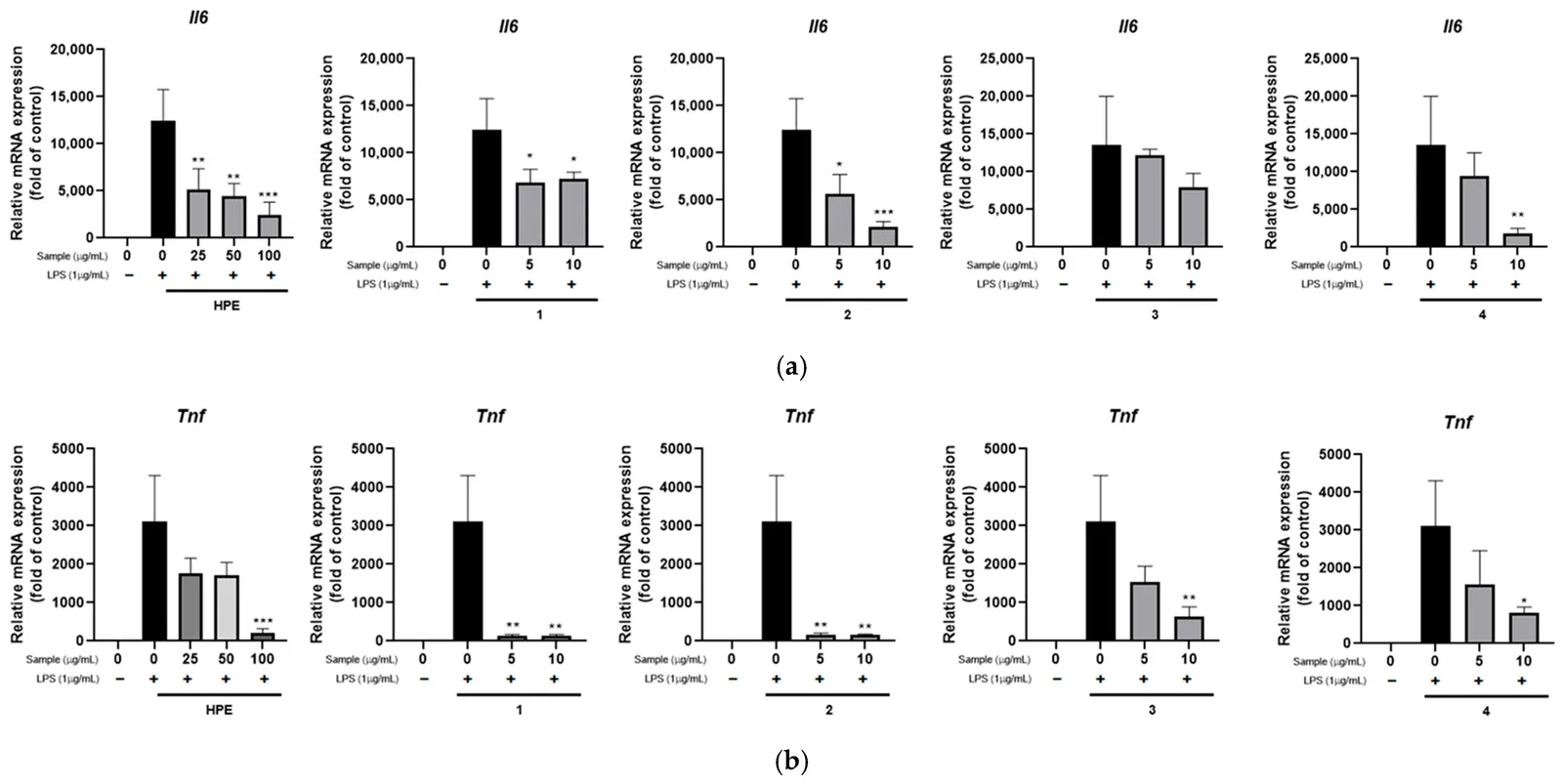
Effects of HPE and compounds **1**–**4** on the mRNA expression of pro-inflammatory cytokines in LPS-stimulated RAW 264.7 macrophages. HPE (25–100 μg/mL) and compounds **1**–**4** (5 and 10 μg/mL) were evaluated for their effects on the mRNA expression of IL-6 (*Il6*) (**a**) and TNF-α (*Tnf*) (**b**) following stimulation with LPS (1 μg/mL). Gene expression levels were normalized to β-actin (*Actb*) and are presented as fold changes relative to the untreated control using the 2^−ΔΔCt^ method. Bar graph representations: white bar, untreated control; black bar, LPS-induced control; grey bar, sample-treated groups in the presence of LPS. Data are presented as mean ± SD. Statistical significance was defined as * *p* < 0.05, ** *p* < 0.01 and *** *p* < 0.001 compared with the LPS-treated group. Compounds: rutin (**1**), hyperoside (**2**), avicularin (**3**), quercetin (**4**).

To further investigate whether the anti-inflammatory activity of HPE and compounds **1**–**4** are associated with transcriptional regulation of these inflammatory mediators, the mRNA expression levels of the pro-inflammatory cytokines, TNF-α and IL-6, were quantified by quantitative real-time PCR (Figure 7).

As expected, LPS stimulation markedly upregulated the mRNA expression of both TNF-α and IL-6 compared with the untreated control group. In contrast, pretreatment with HPE significantly downregulated the expression of both cytokines, with the most pronounced reduction observed at the concentration of 100 μg/mL. Notably, its major active compounds—rutin (**1**), hyperoside (**2**), and quercetin (**4**)—also exerted robust inhibitory effects on the transcriptional activation of both TNF-α and IL-6. Interestingly, avicularin (**3**) exhibited a downregulation of TNF-α mRNA expression, whereas it showed no significant inhibitory effect on IL-6 expression.

Taken together, these results indicate that HPE modulates multiple inflammation-related endpoints in LPS-stimulated macrophages, including NO production, iNOS protein expression, and Tnf/Il6 mRNA expression, validating its therapeutic potential against macrophage-mediated inflammatory diseases at safe, optimized doses.

## 3. Materials and Methods

### 3.1. Plant Materials

Fresh *H. perforatum* samples sourced from Givaudan France Naturals (Reyssouze, France; Product No. EC8418607) were supplied by Maeil Dairies Co., Ltd. (Pyeongtaek, Republic of Korea). *H. perforatum* was collected in China between July and August 2020.

### 3.2. Chemicals and Reagents

Ethanol (EtOH) was purchased from Samchun Pure Chemical (Pyeongtaek, Republic of Korea). HPLC-grade methanol (MeOH), acetonitrile (ACN), and water were obtained from J.T. Baker (Center Valley, PA, USA). Trifluoroacetic acid (TFA; HPLC grade, 99.5 + %; Thermo Fisher Scientific, Waltham, MA, USA; Cat. No. 044630.AY; CAS 76-05-1) and formic acid (FA; 99%, analytical grade; Acros Organics, Geel, Belgium; Cat. No. 270480010; CAS 64-18-6) were used as mobile-phase modifiers. Reference compounds, including rutin (**1**), hyperoside (**2**), avicularin (**3**), quercetin (**4**), and amentoflavone (**5**), were obtained from the Natural Product Institute of Science and Technology, Anseong, Republic of Korea. Dulbecco’s Modified Eagle’s Medium (DMEM), fetal bovine serum (FBS), and penicillin–streptomycin were purchased from Welgene (Gyeongsan, Republic of Korea). LPS and Griess reagent were purchased from Sigma-Aldrich (St. Louis, MO, USA). Dimethyl sulfoxide (DMSO) was obtained from Daejung (Siheung, Republic of Korea).

### 3.3. Instrumentation

Organic solvents were removed using a rotary vacuum evaporator (OSB-2100, Eyela, Tokyo, Japan). LC–MS/MS analyses were performed using an UltiMate 3000 UHPLC system (Thermo Fisher Scientific, Germering, Germany) coupled with a TripleTOF 5600+ high-resolution LC–MS/MS system (SCIEX, Framingham, MA, USA) equipped with a CORTECS C18 column (150 mm × 2.1 mm, 1.6 µm; Waters Corporation, Milford, MA, USA). HPLC analyses were conducted using an Alliance e2695 separations module equipped with a 2998 photodiode array detector (both Waters Corporation, Milford, MA, USA) and an INNO-C18 column (250 mm × 4.6 mm, 5 µm; YMC Co., Ltd., Kyoto, Japan).

### 3.4. Preparation of HPE

The raw material of *H*. *perforatum* was used for the preparation of the extract. The aerial parts of *H. perforatum* were subjected to extraction with 80% EtOH, followed by filtration to remove insoluble plant residues and suspended particles from the extract solution. The 80% EtOH was selected as a hydroethanolic solvent suitable for recovering a broad polarity range of plant secondary metabolites, including phenolic acids, flavonoid glycosides, and relatively less polar flavonoid aglycones. This solvent choice is supported by previous reports showing that ethanol–water mixtures are commonly used for flavonoid extraction and that solvent polarity strongly influences the recovery of phenolic and flavonoid compounds [56,57]. The resulting filtrate was concentrated to reduce the solvent content and then spray-dried to obtain the dried HPE. The extraction yield was 20%.

### 3.5. LC–MS/MS Conditions

The chromatographic column was maintained at 45 °C with a flow rate of 0.25 mL/min. The mobile phases consisted of 0.1% FA in water (A) and 0.1% FA in ACN (B). The gradient elution program was as follows: 3% B from 0–0.5 min, 15% B at 15 min, linearly increased to 100% B by 50 min, maintained at 100% B until 55 min, then returned to 3% B at 55.1 min and held until 60 min for column re-equilibration. Mass spectrometric analysis was performed using an electrospray ionization source operated in both positive and negative ionization modes with information-dependent acquisition. Full-scan mass spectra were acquired over an *m*/*z* range of 100–2000, while MS/MS spectra were acquired over an *m*/*z* range of 30–2000. The source parameters were set as follows: ion source gas 1 (nebulizer gas), 50 psi; ion source gas 2 (heater gas), 50 psi; curtain gas, 25 psi; and source temperature, 500 °C. The ion spray voltage floating was set to −4.5 kV in negative ion mode and +5.5 kV in positive ion mode. The declustering potential was set to +60 V/−60 V. Collision energy was set to +10 V/−10 V for survey MS scans and +35 ± 15 V/−35 ± 15 V for MS/MS acquisition. Nitrogen was used as the collision gas.

### 3.6. Sample and Standard Preparation for LC-MS/MS and HPLC/PDA Analysis

HPE (20 mg) was accurately weighed and dissolved in 1 mL of 80% MeOH (*v*/*v*) for LC–MS/MS and HPLC/PDA analyses. The solutions were then sonicated for 10 min prior to analysis. For preparation of standard solutions, compounds **1**–**5** (1 mg each) were individually dissolved in 1 mL of 80% MeOH and sonicated for 10 min. All samples and standard solutions prepared for chromatographic analysis were filtered through hydrophilic polytetrafluoroethylene syringe filters (PTFE-H, 0.20 µm, 13 mm; Hyundai Micro Co., Ltd., Seoul, Republic of Korea; Cat. No. SH13P020NL) before injection.

### 3.7. Sample and Standard Preparation for HPLC/PDA Analysis

HPE (20 mg) was accurately weighed, dissolved in 1 mL of MeOH, and sonicated for 10 min. For preparation of standard solutions, compounds **1**–**5** (1 mg each) were individually dissolved in 1 mL of MeOH and sonicated for 10 min. All sample and standard solutions prepared for chromatographic analysis were filtered through hydrophilic polytetrafluoroethylene syringe filters (PTFE-H, 0.20 µm, 13 mm; Hyundai Micro Co., Ltd., Seoul, Republic of Korea; Cat. No. SH13P020NL) before injection.

### 3.8. HPLC/PDA Analytical Conditions

Chromatographic separation was performed on the INNO C_18_ column maintained at 30 °C. The mobile phases consisted of 0.1% TFA in water (A) and 0.1% TFA in ACN (B). The gradient program was as follows: 16% B from 0 to 8 min, increased to 20% B at 12 min, 25% B at 22 min, 50% B at 27 min, 90% B at 31 min, and 100% B at 36 min; the system was then returned to 16% B at 38 min and maintained until 54 min for re-equilibration. The injection volume was 5 µL, the flow rate was 1 mL/min, and detection was carried out at 340 nm.

### 3.9. Calibration and Quantification

Standard solutions were serially diluted to concentrations ranging from 15.625 to 500 µg/mL. Calibration curves were constructed by plotting peak area (Y, mAU) against concentration (X, µg/mL), and linearity was evaluated using the correlation coefficient (*R*^2^). All analyses were performed in triplicate.

### 3.10. Cell Viability Assay in Macrophages

The murine macrophage cell line RAW 264.7 was obtained from the Korean Cell Line Bank (KCLB, Seoul, Republic of Korea). Cells were cultured in DMEM supplemented with 10% FBS and 1% penicillin–streptomycin at 37 °C in a humidified incubator containing 5% CO_2_. The culture medium was replaced every 2 days. When the cells reached approximately 80–90% confluence, they were washed with phosphate-buffered saline (PBS) and harvested using a cell scraper. Cell viability was evaluated using an MTT assay to assess the cytotoxic effects of the samples [58]. Cells were seeded into 96-well plates at a density of 2.5 × 10^4^ cells/mL and allowed to stabilize for 24 h prior to treatment with HPE (10, 25, 50, and 100 μg/mL) or compounds **1**–**5** (1, 2.5, and 10 μg/mL). After 24 h of treatment, the culture medium was removed, and MTT solution (Thermo Fisher Scientific, Waltham, MA, USA) was added to each well, followed by incubation for 4 h. The resulting formazan crystals were dissolved in DMSO, and absorbance was measured at 560 nm using a microplate reader (Thermo Fisher Scientific, Waltham, MA, USA).

### 3.11. NO Assay in LPS-Stimulated Macrophages

NO production was evaluated using a Griess assay in LPS-stimulated RAW 264.7 macrophages [59]. Cells were seeded into 96-well plates at a density of 1 × 10^6^ cells/mL and incubated for 24 h. The cells were then treated with various concentrations of HPE (0, 5, 10, 25, 50, and 100 μg/mL) or compounds **1**–**5** (0.5, 1, 2.5, 5, and 10 μg/mL). After 2 h of sample treatment, LPS (1 μg/mL) was added to induce an inflammatory response. Following an additional 24 h incubation period, 100 μL of culture supernatant was mixed with an equal volume of Griess reagent and incubated at room temperature for 15 min. Absorbance was subsequently measured at 560 nm using a microplate reader.

### 3.12. Western Blotting

Western blot analysis was performed using protein extracts obtained from RAW 264.7 cells [60]. Following the respective treatments, cells were lysed using PRO-PREP™ protein extraction solution (iNtRON Biotechnology, Seongnam, Republic of Korea) according to the manufacturer’s instructions. Protein concentrations were determined using the Bradford protein assay with bovine serum albumin (BSA) as the standard. Equal amounts of protein were mixed with 2× Laemmli sample buffer containing β-mercaptoethanol and denatured at 100 °C for 10 min. The proteins were separated by 10% sodium dodecyl sulfate–polyacrylamide gel electrophoresis (SDS-PAGE) and transferred onto 0.2 μm polyvinylidene fluoride (PVDF) membranes (Millipore, Billerica, MA, USA). The membranes were blocked with 5% BSA for 1 h at room temperature and subsequently incubated overnight at 4 °C with an anti-iNOS primary antibody (Cell Signaling Technology, Danvers, MA, USA; Cat. #13120; 1:1000). After washing with Tris-buffered saline containing 0.1% Tween-20 (TBS-T), the membranes were incubated with an HRP-conjugated goat anti-rabbit IgG secondary antibody for 1 h at room temperature. Protein bands were visualized using an enhanced chemiluminescence (ECL) substrate and detected with a ChemiDoc Imaging System (Bio-Rad, Hercules, CA, USA). Band intensities were quantified using ImageJ software (Version 1.53, National Institutes of Health, Bethesda, MD, USA) and normalized to β-actin.

### 3.13. Quantitative Real-Time PCR (qPCR)

Total RNA was isolated from RAW 264.7 cells using TRIzol^®^ reagent (Invitrogen™, Thermo Fisher Scientific, Waltham, MA, USA) in accordance with the manufacturer’s protocol. The quantity and purity of the extracted RNA were assessed by measuring the absorbance at 260 and 280 nm with a NanoDrop spectrophotometer (Thermo Fisher Scientific, Waltham, MA, USA). For cDNA synthesis, 1 μg of total RNA was reverse-transcribed using AccuPower^®^ RT PreMix (Bioneer, Daejeon, Republic of Korea). The resulting cDNA was stored at −20 °C until subsequent analysis. qPCR was carried out using a Bio-Rad CFX96 Real-Time PCR Detection System (Bio-Rad, Hercules, CA, USA) with SYBR Green PCR Master Mix. Each reaction was prepared in a final volume of 20 μL. Amplification was initiated with a denaturation step at 95 °C, followed by 40 amplification cycles consisting of denaturation at 95 °C and annealing/extension at 60 °C. Gene expression levels were normalized to the housekeeping gene *Actb* (β-actin), and relative mRNA expression was determined using the comparative 2^−ΔΔCt^ method. The primer sequences used in this study are presented in Table 4.

### 3.14. Statistical Analysis

Results are presented as the mean ± standard deviation (SD). Statistical analyses were performed using GraphPad Prism 9 (GraphPad Software, San Diego, CA, USA). Differences among groups were evaluated by one-way analysis of variance (ANOVA) followed by Tukey’s post hoc test. Statistical significance was defined as *p* < 0.05.

## 4. Conclusions

In the present study, HPE was chemically characterized and evaluated for its inhibitory effects on NO production in LPS-stimulated RAW 264.7 macrophages. LC–MS/MS profiling demonstrated that HPE possessed a flavonoid-rich phenolic composition, with flavonoid-related metabolites representing the largest proportion of the tentative annotation profile. Multiple quercetin-derived glycosides and conjugates were detected, indicating extensive structural diversification of the quercetin-type flavonol scaffold within the extract. Subsequent HPLC/PDA analysis provided standard-based quantitative data for selected flavonoid constituents. Among the quantified markers, hyperoside (**2**) was identified as the most abundant compound, followed by rutin (**1**), quercetin (**4**), and avicularin (**3**), whereas amentoflavone (**5**) was detected only at trace levels. Functional assays demonstrated that HPE and its selected quantified flavonoid markers (compounds **1**–**4**) suppressed LPS-induced NO production, whereas compound **5** showed no significant effect. At the molecular level, HPE and its selected flavonoid markers markedly suppressed iNOS protein expression and downregulated the mRNA expression of the pro-inflammatory cytokines, *Tnf* and *Il6*. Interestingly, avicularin (**3**) exhibited a distinct regulatory profile by reducing *Tnf* mRNA expression, whereas its effect on *Il6* mRNA expression was not significant under the tested conditions. These findings suggest that HPE and its flavonoids have anti-inflammatory potential in LPS-stimulated macrophages, which is associated, at least in part, with downregulation of iNOS protein expression and modulation of pro-inflammatory cytokine mRNA expression. However, the estimated concentrations of the quantified flavonoid markers present in HPE at the biologically active extract concentration were lower than the concentrations at which the isolated compounds exhibited significant NO inhibitory effects. Therefore, the extract-level response could not be directly assigned to any single quantified marker, and additive or synergistic interactions among constituents were not directly demonstrated in this study. Although further mechanistic studies are warranted to elucidate the precise upstream signaling pathways, HPE may serve as a chemically characterized plant extract with inflammation-modulatory potential, warranting further investigation of its active constituents, constituent interactions, and broader biological relevance.

## Figures and Tables

**Table 1:** Tentatively identified compounds and proposed structures in HPE based on LC–MS/MS analysis in negative ion mode.

Retention Time (min)	Adducts	Calculated *m*/*z*	Observed *m*/*z*	Δ ppm	Molecular Formula	Tentative Identification
5.54	[M − H]^−^	153.0193	153.0203	+6.58	C_7_H_6_O_4_	Protocatechuic acid ^2^
7.53	[M − H]^−^	353.0878	353.0900	+6.23	C_16_H_18_O_9_	Neochlorogenic acid ^3^
7.81	[M − H]^−^	137.0244	137.0253	+6.53	C_7_H_6_O_3_	Protocatechuic aldehyde ^2^
9.35	[M − H]^−^	577.1351	577.1396	+7.72	C_30_H_26_O_12_	Procyanidin B1 ^3^
9.70	[M − H]^−^	337.0928	337.0948	+5.78	C_16_H_18_O_8_	5-*O*-Coumaroylquinic acid ^3^
9.86	[M − H]^−^	577.1351	577.1400	+8.54	C_30_H_26_O_12_	Procyanidin B3 ^3^
10.26	[M − H]^−^	289.0717	289.0735	+6.06	C_15_H_14_O_6_	Catechin ^3^
10.45	[M − H]^−^	355.1034	355.1055	+5.91	C_16_H_20_O_9_	1-*O*-Feruloylglucose ^3^
10.78	[M − H]^−^	353.0878	353.0896	+5.15	C_16_H_18_O_9_	Chlorogenic acid ^1^
11.48	[M − H]^−^	353.0878	353.0892	+4.11	C_16_H_18_O_9_	Cryptochlorogenic acid ^1^
13.70	[M − H]^−^	289.0717	289.0732	+5.05	C_15_H_14_O_6_	Epicatechin ^1^
13.79	[M − H]^−^	337.0928	337.0946	+5.25	C_16_H_18_O_8_	3-*O*-Coumaroylquinic acid ^3^
14.03	[M − H]^−^	337.0928	337.0943	+4.31	C_16_H_18_O_8_	4-*O*-Coumaroylquinic acid ^1^
14.80	[M − H]^−^	421.0776	421.0796	+4.82	C_19_H_18_O_11_	Mangiferin ^2^
14.83	[M − H]^−^	163.0400	163.0408	+4.51	C_9_H_8_O_3_	*p*-Coumaric acid ^1^
16.20	[M − H]^−^	337.0928	337.0946	+5.23	C_16_H_18_O_8_	1-*O*-Coumaroylquinic acid ^1^
17.07	[M − H]^−^	479.0831	479.0859	+5.98	C_21_H_20_O_13_	Myricetin 3-*O*-galactoside ^3^
17.44	[M − H]^−^	479.0831	479.0858	+5.80	C_21_H_20_O_13_	Myricetin 3-*O*-glucoside ^3^
19.57	[M − H]^−^	609.1461	609.1515	+8.92	C_27_H_30_O_16_	Rutin ^2^
19.69	[M − H]^−^	477.0674	477.0709	+7.28	C_21_H_18_O_13_	Quercetin 3-*O*-glucuronide ^3^
19.87	[M − H]^−^	463.0881	463.0912	+6.67	C_21_H_20_O_12_	Hyperoside ^2^
20.77	[M − H]^−^	447.0932	447.0958	+5.64	C_21_H_20_O_11_	Cynaroside ^3^
20.91	[M − H]^−^	593.1511	593.1553	+7.02	C_27_H_30_O_15_	Nicotiflorin ^3^
20.99	[M − H]^−^	433.0776	433.0800	+5.52	C_20_H_18_O_11_	Avicularin ^2^
21.14	[M − H]^−^	463.0881	463.0904	+4.78	C_21_H_20_O_12_	Spiraeoside ^3^
21.35	[M − H]^−^	447.0932	447.0956	+5.24	C_21_H_20_O_11_	Quercitrin ^3^
21.91	[M − H]^−^	515.1194	515.1233	+7.50	C_25_H_24_O_12_	4,5-di-*O*-Caffeoylquinic acid ^3^
22.09	[M − H]^−^	505.0987	505.1024	+7.19	C_23_H_22_O_13_	Quercetin 3-*O*-glucose-6″-acetate ^3^
24.40	[M − H]^−^	301.0353	301.0370	+5.38	C_15_H_10_O_7_	Quercetin ^2^
24.44	[M − H]^−^	285.0404	285.0417	+4.46	C_15_H_10_O_6_	Luteolin ^2^
26.57	[M − H]^−^	285.0404	285.0415	+3.75	C_15_H_10_O_6_	Kaempferol ^2^
27.54	[M − H]^−^	537.0827	537.0866	+7.27	C_30_H_18_O_10_	Amentoflavone ^2^
29.89	[M − H]^−^	327.0874	327.0888	+4.36	C_18_H_16_O_6_	3-Hydroxy-7,2′,4′-trimethoxyflavone ^3^
31.12	[M − H]^−^	257.0455	257.0461	+2.27	C_14_H_10_O_5_	Isogentisin ^2^

Superscript numbers indicate MSI confidence levels: ^1^ MSI Level 1; ^2^ MSI Level 2; ^3^ MSI Level 3.

**Table 2:** Tentatively identified compounds and proposed structures in HPE based on LC–MS/MS analysis in positive ion mode.

Retention Time (min)	Adducts	Calculated *m*/*z*	Observed *m*/*z*	Δ ppm	Molecular Formula	Tentative Identification
7.53	[M + H]^+^	355.1023	355.1023	−0.01	C_16_H_18_O_9_	Neochlorogenic acid ^3^
10.26	[M + H]^+^	291.0863	291.0865	+0.87	C_15_H_14_O_6_	Catechin ^3^
10.78	[M + H]^+^	355.1023	355.1025	+0.47	C_16_H_18_O_9_	Chlorogenic acid ^1^
11.48	[M + H]^+^	355.1023	355.1025	+0.48	C_16_H_18_O_9_	Cryptochlorogenic acid ^1^
13.70	[M + H]^+^	291.0863	291.0862	−0.08	C_15_H_14_O_6_	Epicatechin ^1^
13.79	[M + H]^+^	339.1074	339.1073	−0.37	C_16_H_18_O_8_	3-*O*-Coumaroylquinic acid ^3^
14.80	[M + H]^+^	423.0921	423.0910	−2.68	C_19_H_18_O_11_	Mangiferin ^2^
19.57	[M + H]^+^	611.1606	611.1610	+0.55	C_27_H_30_O_16_	Rutin ^2^
19.69	[M + H]^+^	479.0820	479.0816	−0.80	C_21_H_18_O_13_	Quercetin 3-*O*-glucuronide ^3^
19.87	[M + H]^+^	465.1027	465.1029	+0.51	C_21_H_20_O_12_	Hyperoside ^2^
20.67	[M + H]^+^	435.0921	435.0920	−0.34	C_20_H_18_O_11_	Guaiaverin ^3^
20.77	[M + H]^+^	449.1078	449.1074	−0.98	C_21_H_20_O_11_	Cynaroside ^3^
20.99	[M + H]^+^	435.0921	435.0926	+5.52	C_20_H_18_O_11_	Avicularin ^2^
21.35	[M + H]^+^	449.1078	449.1079	+0.15	C_21_H_20_O_11_	Quercitrin ^3^
22.09	[M + H]^+^	507.1133	507.1132	−0.12	C_23_H_22_O_13_	Quercetin-3-*O*-glucose-6″-acetate ^3^
24.40	[M + H]^+^	303.0499	303.0499	−1.35	C_15_H_10_O_7_	Quercetin ^2^
24.44	[M + H]^+^	287.0550	287.0548	−0.60	C_15_H_10_O_6_	Luteolin ^2^
24.48	[M + H]^+^	593.1864	593.1857	−1.18	C_28_H_32_O_14_	Linarin ^3^
27.54	[M + H]^+^	539.0972	539.0963	−2.39	C_30_H_18_O_10_	Amentoflavone ^2^
29.89	[M + H]^+^	329.1019	329.1017	−0.72	C_18_H_16_O_6_	3-Hydroxy-7,2′,4′-trimethoxyflavone ^3^

Superscript numbers indicate MSI confidence levels: ^1^ MSI Level 1; ^2^ MSI Level 2; ^3^ MSI Level 3.

**Table 3:** Calibration curves and quantification for compounds **1**–**5**.

Compound	Retention Time (min)	Calibration Equation	*R* ^2^	Content (mg/g ext.)
**1**	16.6	y = 5719.1 x + 13,329	0.9995	9.18 ± 0.17
**2**	17.5	y = 8293.2 x + 3054.8	0.9998	12.44 ± 0.23
**3**	21.3	y = 8046.6 x + 738.56	0.9999	5.55 ± 0.13
**4**	29.4	y = 10,210 x + 11,623	0.9997	6.64 ± 0.08
**5**	31.3	-	-	tr

y, peak area; x, concentration of the standard (µg/mL); *R*^2^, correlation coefficient for six calibration data points (*n* = 3); tr, trace level. Compounds: rutin (**1**), hyperoside (**2**), avicularin (**3**), quercetin (**4**), and amentoflavone (**5**).

**Table 4:** Primer sequences of target genes for quantitative real-time reverse transcription polymerase chain reaction analysis.

Gene	Forward Primer (5′ → 3′)	Reverse Primer (5′ → 3′)
*A* *ctb*	CCAACCGTGAAAAGATGACC	CCAGAGGCATACAGGGACAG
*Il6*	TAGTCCTTCCTACCCCAATTTCC	TTGGTCCTTAGCCACTCCTTC
*Tnf*	GCAACTGTTCCTGAACTCAACT	ATCTTTTGGGGTCCGTCAACT

## Data Availability

The original contributions presented in this study are included in the article/Supplementary Materials; further inquiries can be directed to the corresponding author.

## Supplementary Materials

The following supporting information can be downloaded at: https://www.mdpi.com/article/10.3390/ph19081136/s1. Figure S1. Representative MS/MS spectral mirror plots of the tentatively identified compounds in HPE listed in Table 1. The upper blue spectra indicate the experimentally observed MS/MS spectra, whereas the lower red spectra indicate the corresponding reference spectra. The spectra support the tentative LC–MS/MS annotations of the following compounds: protocatechuic acid (a), neochlorogenic acid (b), protocatechuic aldehyde (c), procyanidin B1 (d), 5-*O*-coumaroylquinic acid (e), procyanidin B3 (f), catechin (g), 1-*O*-feruloylglucose (h), chlorogenic acid (i), cryptochlorogenic acid (j), epicatechin (k), 3-*O*-coumaroylquinic acid (l), 4-*O*-coumaroylquinic acid (m), mangiferin (n), *p*-coumaric acid (o), 1-*O*-coumaroylquinic acid (p), myricetin 3-*O*-galactoside (q), myricetin 3-*O*-glucoside (r), rutin (s), quercetin 3-*O*-glucuronide (t), hyperoside (u), cynaroside (v), nicotiflorin (w), avicularin (x), spiraeoside (y), quercitrin (z), 4,5-di-*O*-caffeoylquinic acid (aa), quercetin 3-*O*-glucose-6″-acetate (ab), quercetin (ac), luteolin (ad), kaempferol (ae), amentoflavone (af), 3-hydroxy-7,2′,4′-trimethoxyflavone (ag), and isogentisin (ah); Figure S2. Representative MS/MS spectral mirror plots of the tentatively identified compounds in HPE listed in Table 2. The upper blue spectra indicate the experimentally observed MS/MS spectra, whereas the lower red spectra indicate the corresponding reference spectra. The spectra support the tentative LC–MS/MS annotations of the following compounds: neochlorogenic acid (a), catechin (b), chlorogenic acid (c), cryptochlorogenic acid (d), epicatechin (e), 3-*O*-coumaroylquinic acid (f), mangiferin (g), rutin (h), quercetin 3-*O*-glucuronide (i), hyperoside (j), guaiaverin (k), cynaroside (l), avicularin (m), quercitrin (n), quercetin 3-*O*-glucose-6″-acetate (o), quercetin (p), luteolin (q), linarin (r), and 3-hydroxy-7,2′,4′-trimethoxyflavone (s); Figure S3. HPE and compounds **1**–**4** original Western blot band images.
