# Supplementary material for: LC–MS/MS Chemical Profiling and HPLC/PDA Quantification of Hypericum perforatum Extract and Its Anti-Inflammatory Properties
Source: Pharmaceuticals (Basel). 2026 Jul 23;19(8):1136. doi: 10.3390/ph19081136 (PMC13516343; doi:10.3390/ph19081136)
Supplement: Supplementary file 1 [file pharmaceuticals-19-01136-s001.zip › pharmaceuticals-4389880 SM1.pdf]

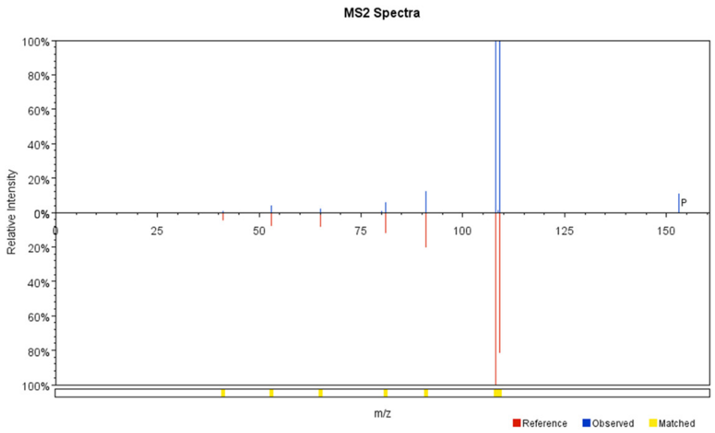
MS2 Spectra
Relative Intensity
m/z
Reference
Observed
Matched

(a)

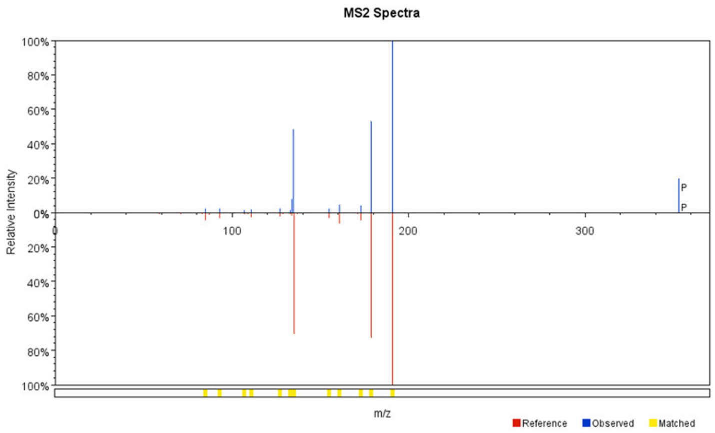
MS2 Spectra
Relative Intensity
m/z
Reference
Observed
Matched

(b)

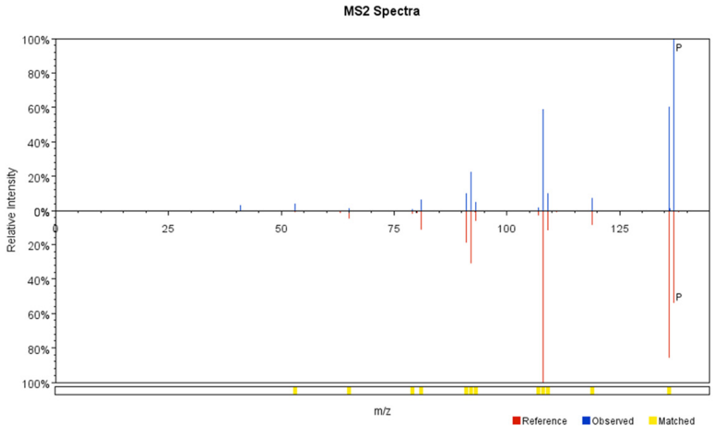
MS2 Spectra
Relative Intensity
m/z
Reference
Observed
Matched

(c)

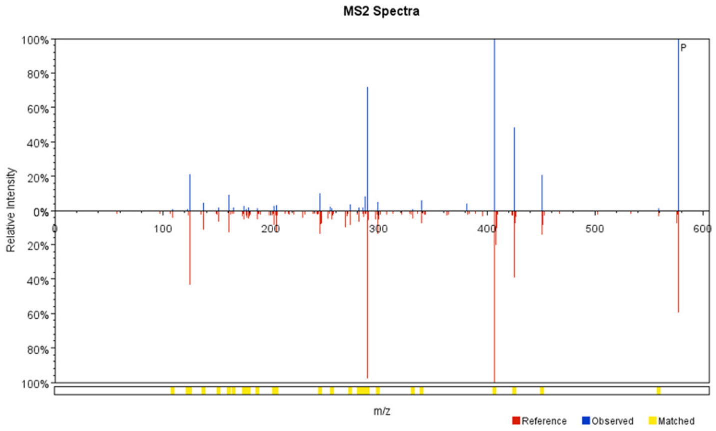
MS2 Spectra
Relative Intensity
m/z
Reference
Observed
Matched

(d)

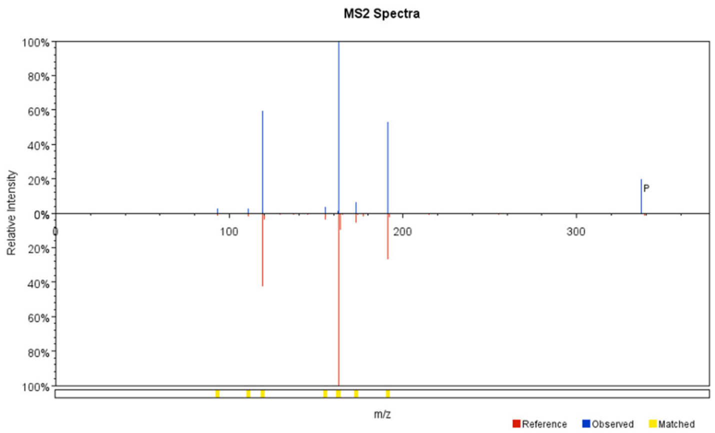
MS2 Spectra
Relative Intensity
m/z
Reference
Observed
Matched
P

(e)

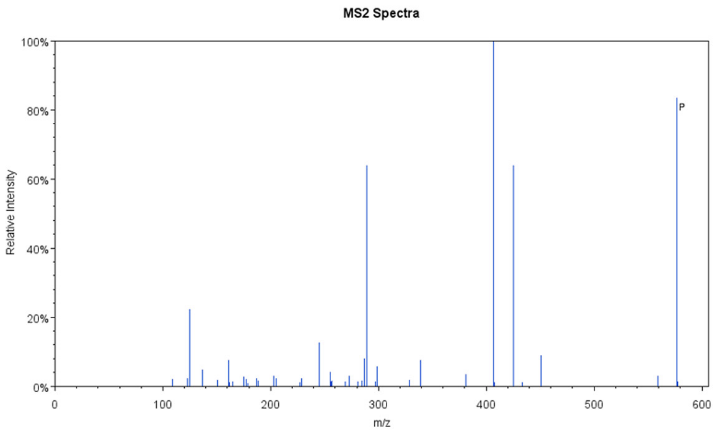
MS2 Spectra
Relative Intensity
m/z
P

(f)

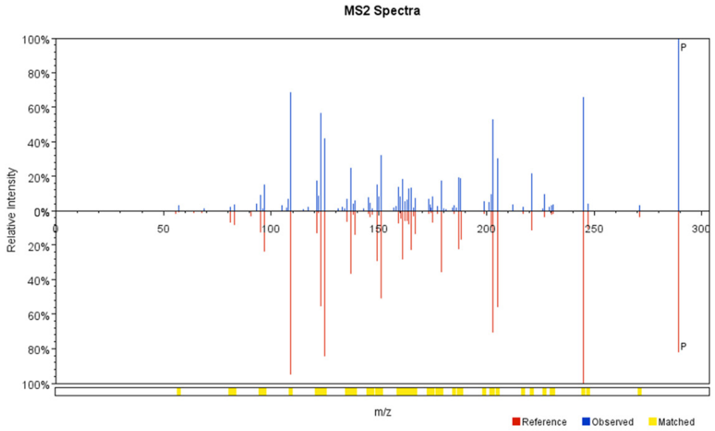
MS2 Spectra
Relative Intensity
m/z
Reference
Observed
Matched
P

(g)

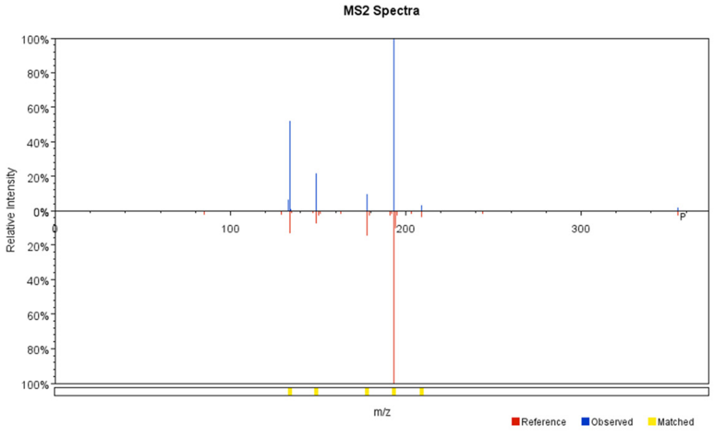
MS2 Spectra
Relative Intensity
m/z
Reference
Observed
Matched
P

(h)

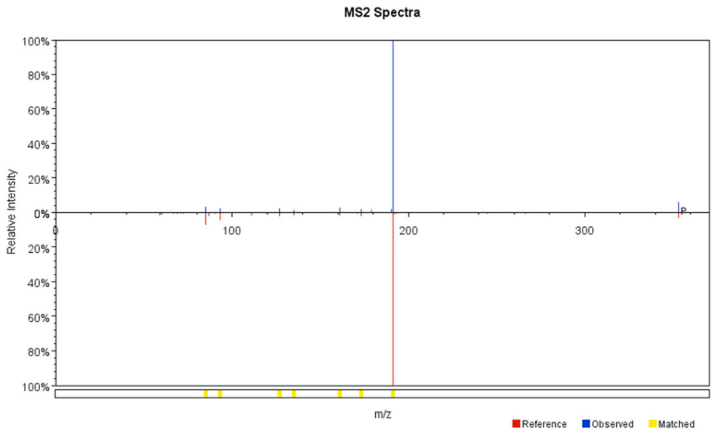
MS2 Spectra
Relative Intensity
m/z
Reference
Observed
Matched
P

(i)

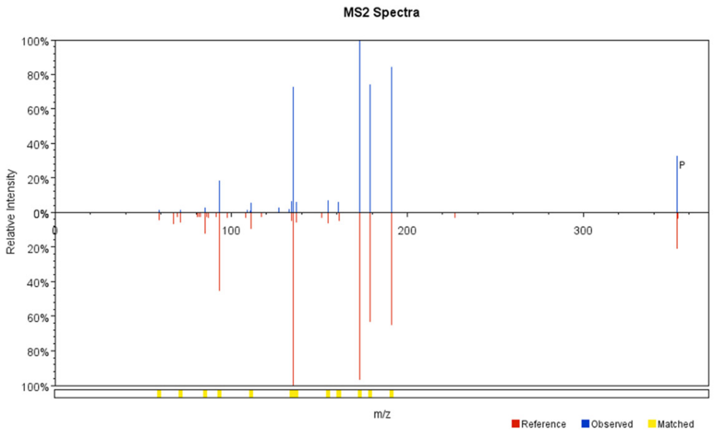
MS2 Spectra
Relative Intensity
m/z
Reference
Observed
Matched
P

(j)

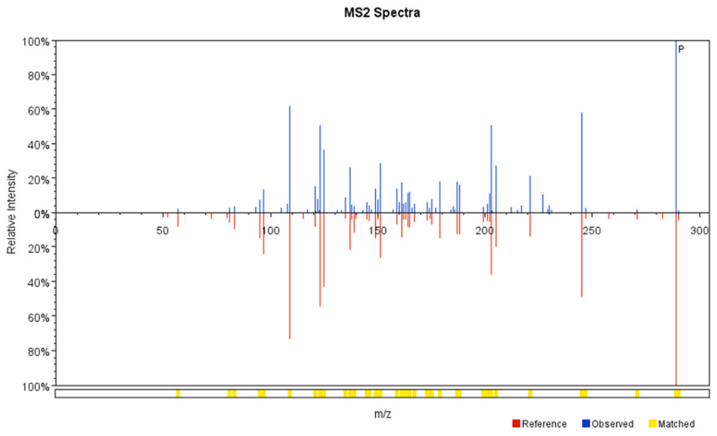
MS2 Spectra
Relative Intensity
m/z
Reference
Observed
Matched
P

(k)

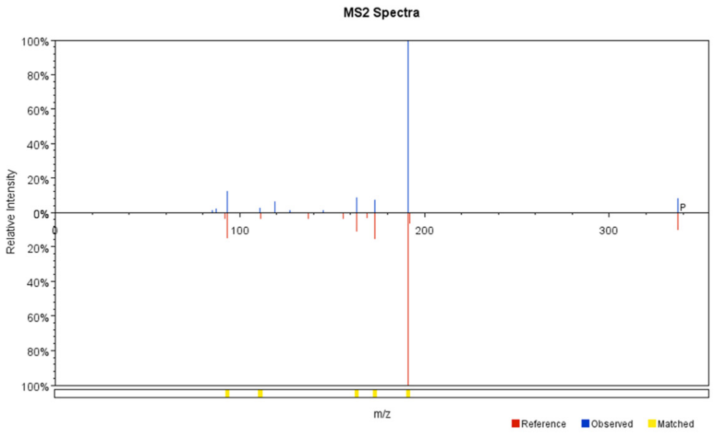
MS2 Spectra
Relative Intensity
m/z
Reference
Observed
Matched
P

(l)

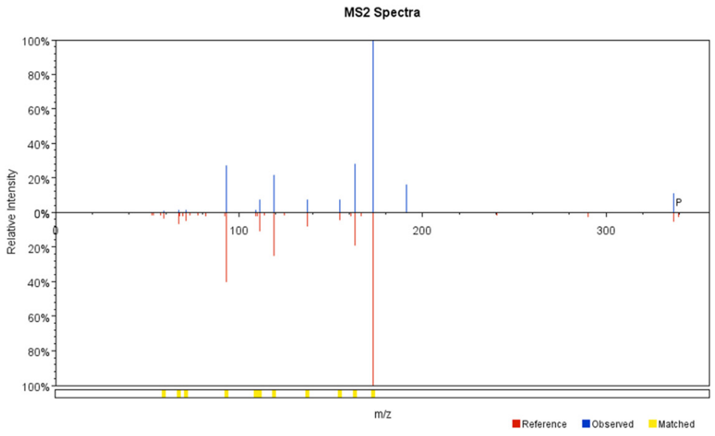
MS2 Spectra
Relative Intensity
m/z
Reference
Observed
Matched

(m)

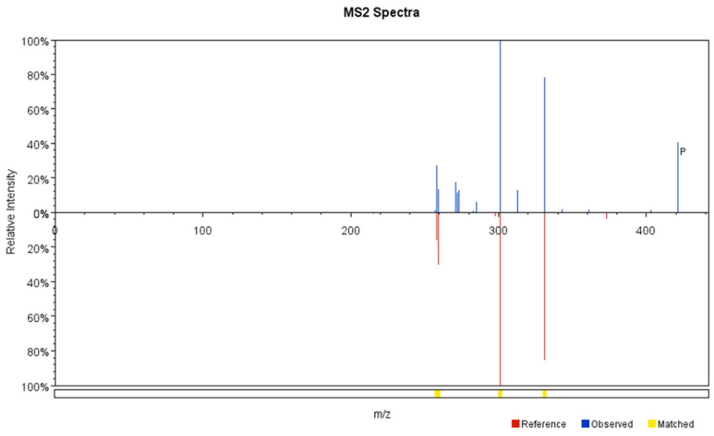
MS2 Spectra
Relative Intensity
m/z
Reference
Observed
Matched

(n)

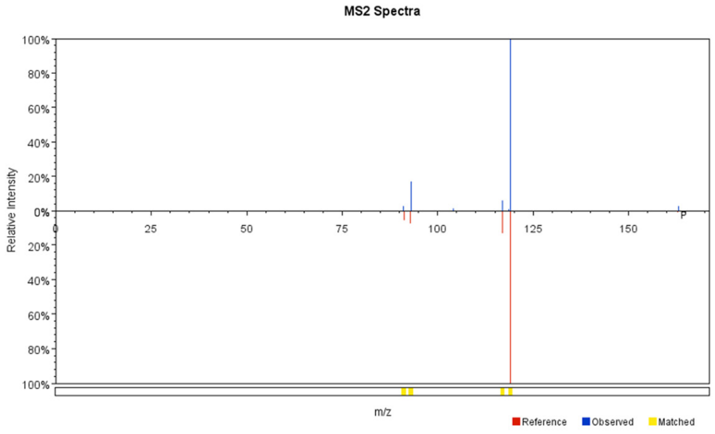
MS2 Spectra
Relative Intensity
m/z
Reference
Observed
Matched

(o)

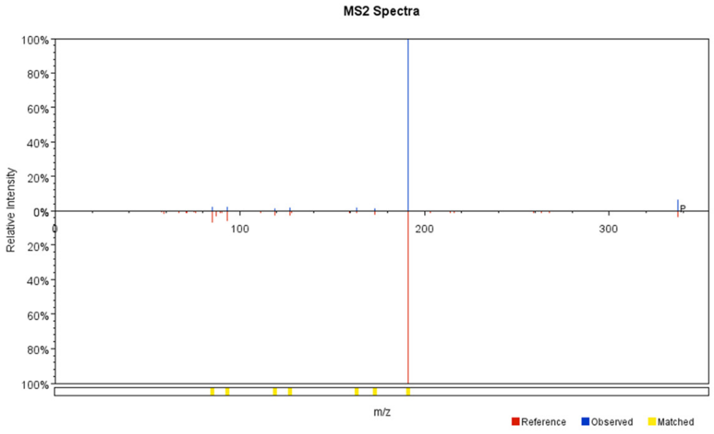
MS2 Spectra
Relative Intensity
m/z
Reference
Observed
Matched

(p)

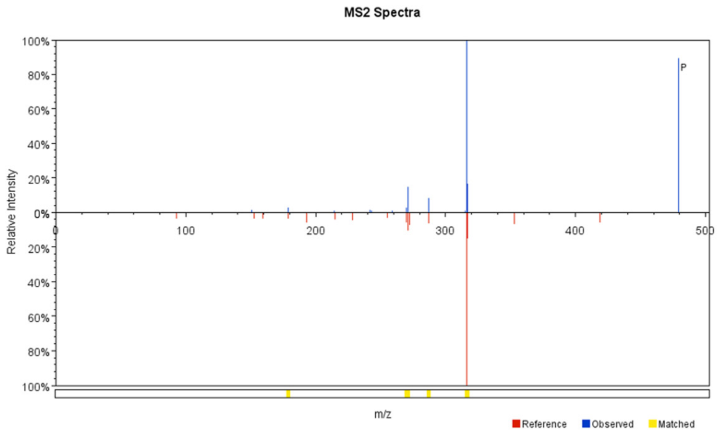
MS2 Spectra
Relative Intensity
m/z
P
Reference
Observed
Matched

(q)

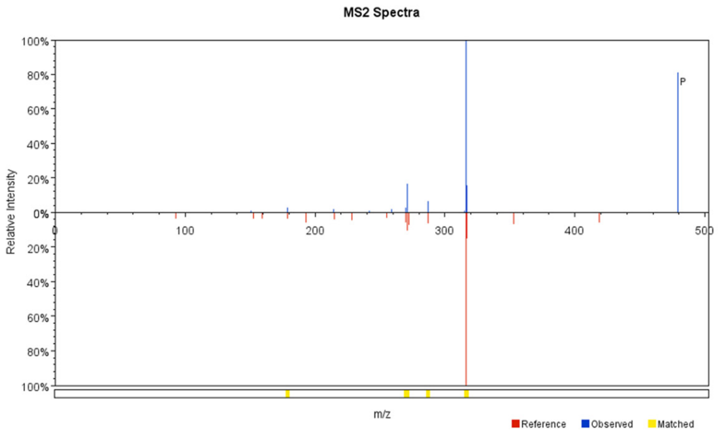
MS2 Spectra
Relative Intensity
m/z
P
Reference
Observed
Matched

(r)

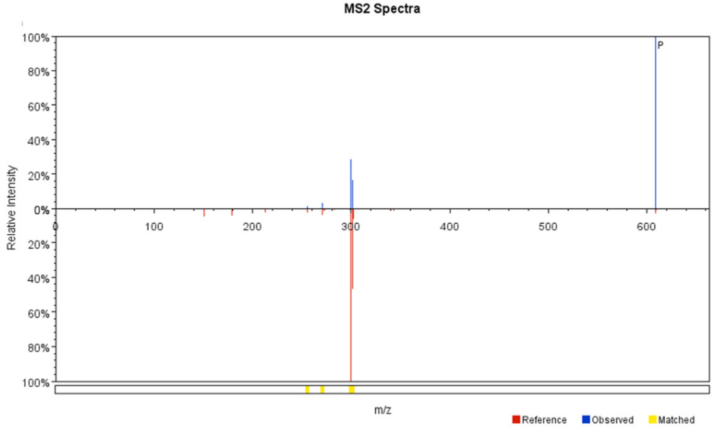
MS2 Spectra
Relative Intensity
m/z
P
Reference
Observed
Matched

(s)

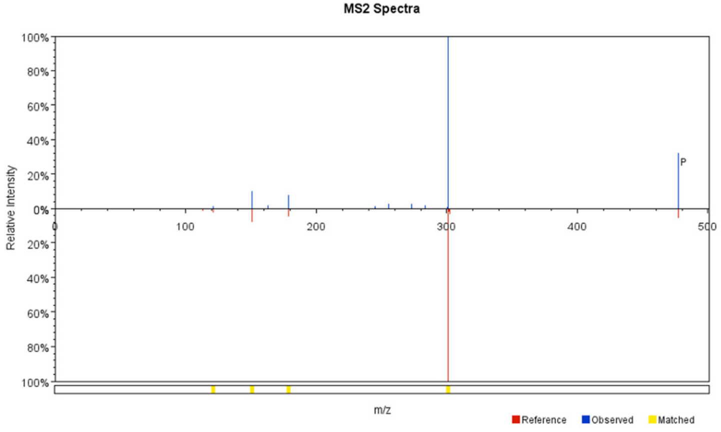
MS2 Spectra
Relative Intensity
m/z
P
Reference
Observed
Matched

(t)

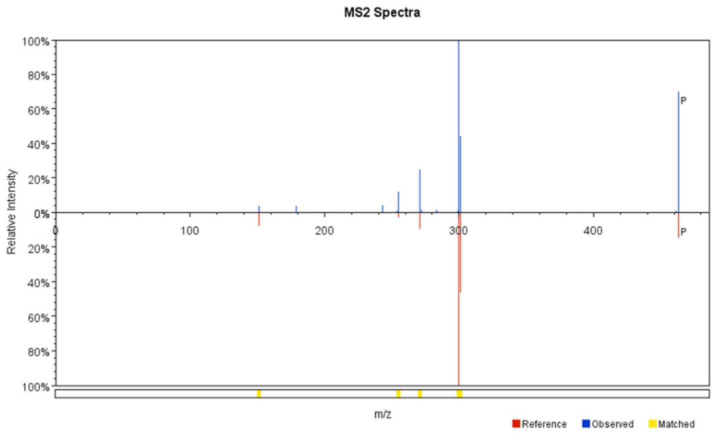
MS2 Spectra
Relative Intensity
m/z
Reference
Observed
Matched

(**u**)

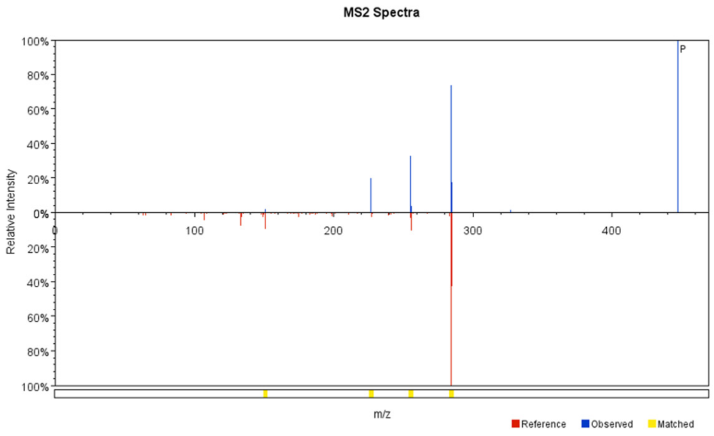
MS2 Spectra
Relative Intensity
m/z
Reference
Observed
Matched

(**v**)

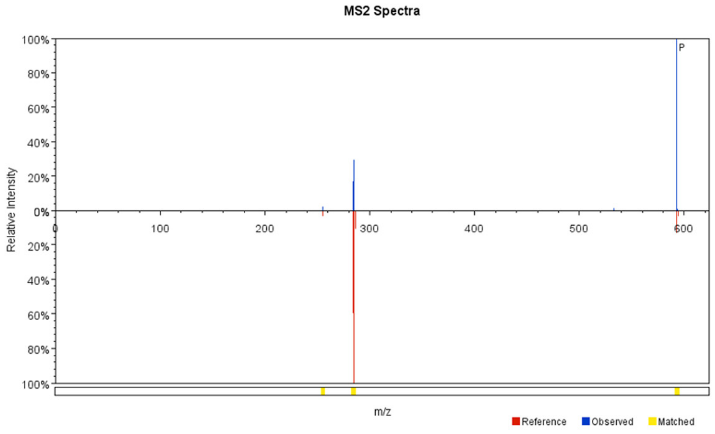
MS2 Spectra
Relative Intensity
m/z
Reference
Observed
Matched

(**w**)

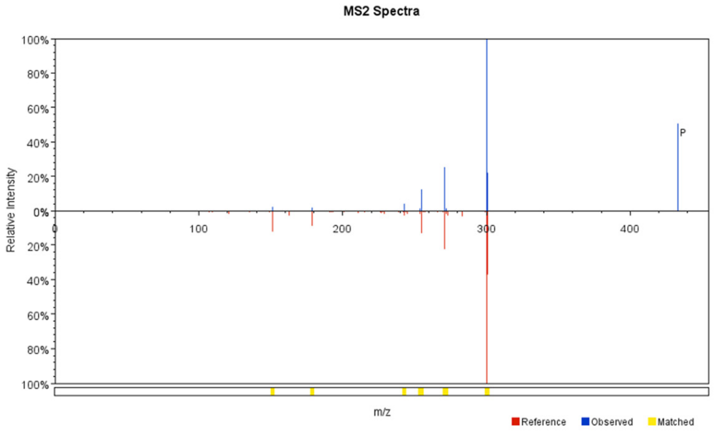
MS2 Spectra
Relative Intensity
m/z
Reference
Observed
Matched

(**x**)

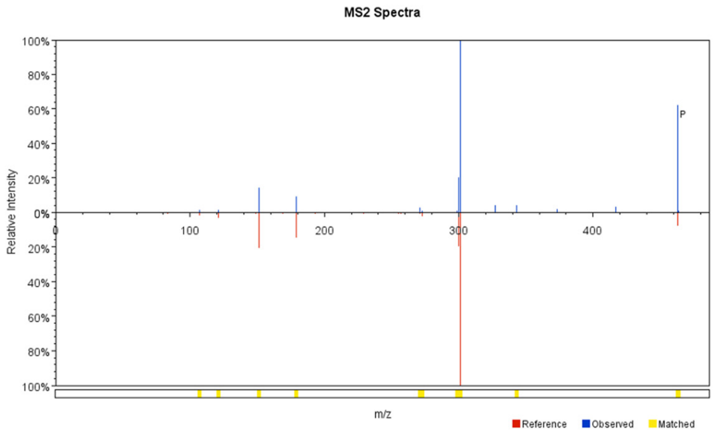
MS2 Spectra
Relative Intensity
100%
80%
60%
40%
20%
0%
20%
40%
60%
80%
100%
0
100
200
300
400
P
m/z
Reference
Observed
Matched

(y)

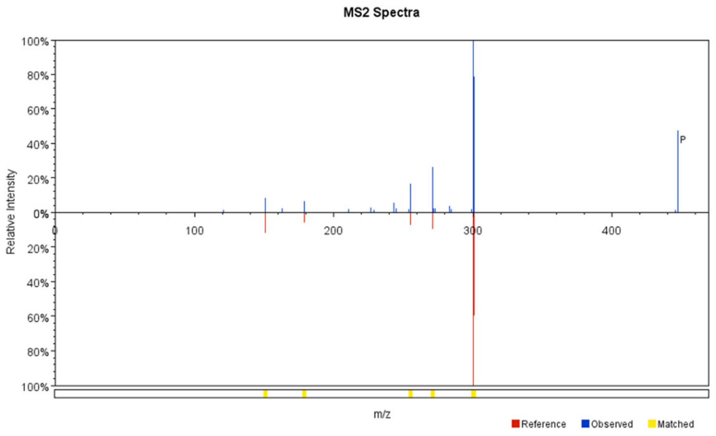
MS2 Spectra
Relative Intensity
100%
80%
60%
40%
20%
0%
20%
40%
60%
80%
100%
0
100
200
300
400
P
m/z
Reference
Observed
Matched

(z)

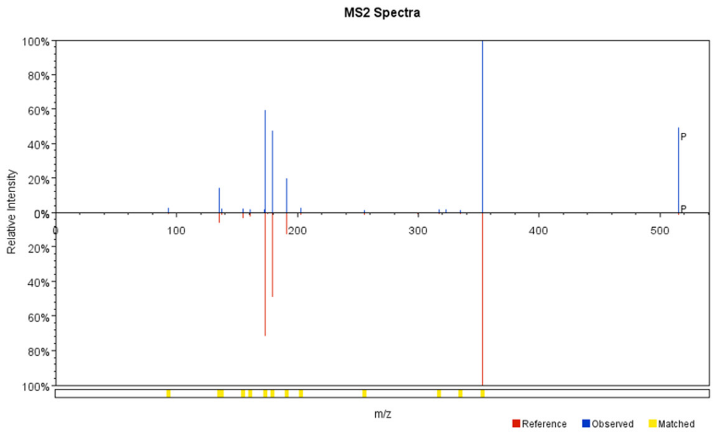
MS2 Spectra
Relative Intensity
100%
80%
60%
40%
20%
0%
20%
40%
60%
80%
100%
0
100
200
300
400
500
P
P
m/z
Reference
Observed
Matched

(aa)

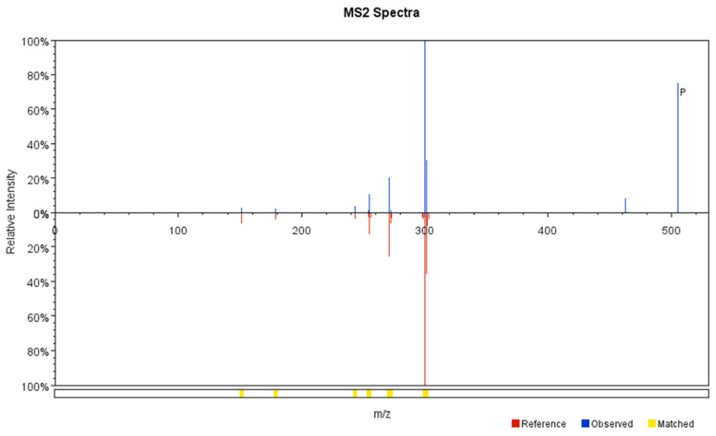
MS2 Spectra
Relative Intensity
100%
80%
60%
40%
20%
0%
20%
40%
60%
80%
100%
0
100
200
300
400
500
P
m/z
Reference
Observed
Matched

(ab)

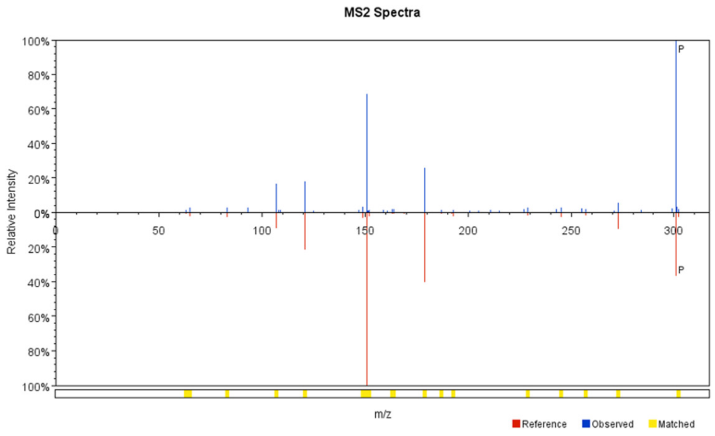
MS2 Spectra
Relative Intensity
100%
80%
60%
40%
20%
0%
20%
40%
60%
80%
100%
0
50
100
150
200
250
300
P
P
m/z
Reference
Observed
Matched

(ac)

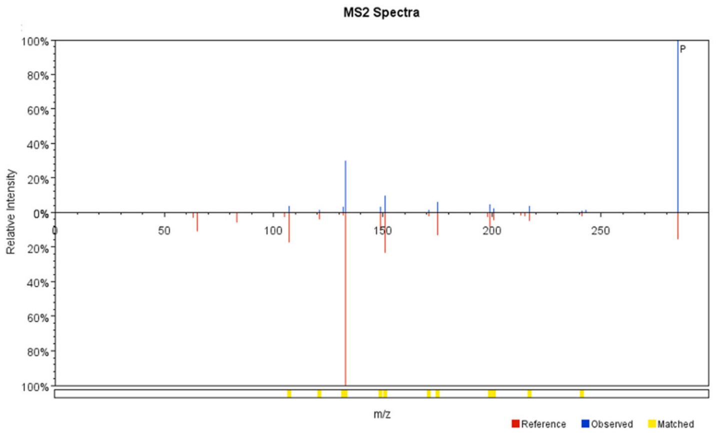
MS2 Spectra
Relative Intensity
100%
80%
60%
40%
20%
0%
20%
40%
60%
80%
100%
0
50
100
150
200
250
P
m/z
Reference
Observed
Matched

(ad)

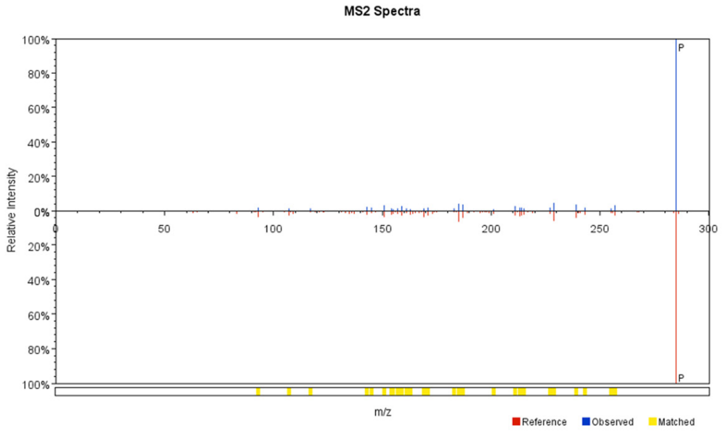
MS2 Spectra
Relative Intensity
100%
80%
60%
40%
20%
0%
20%
40%
60%
80%
100%
0
50
100
150
200
250
300
P
P
m/z
Reference
Observed
Matched

(ae)

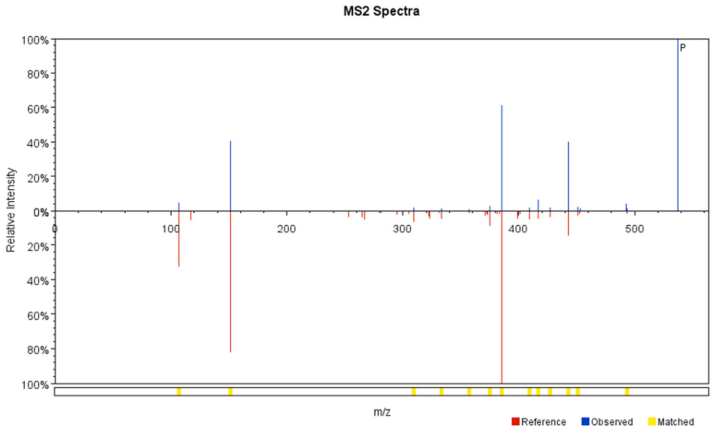
MS2 Spectra
Relative Intensity
100%
80%
60%
40%
20%
0%
20%
40%
60%
80%
100%
0
100
200
300
400
500
P
m/z
Reference
Observed
Matched

(af)

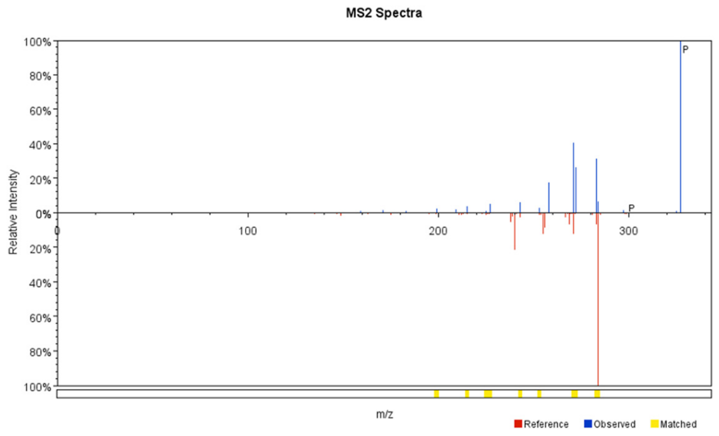

(ag)

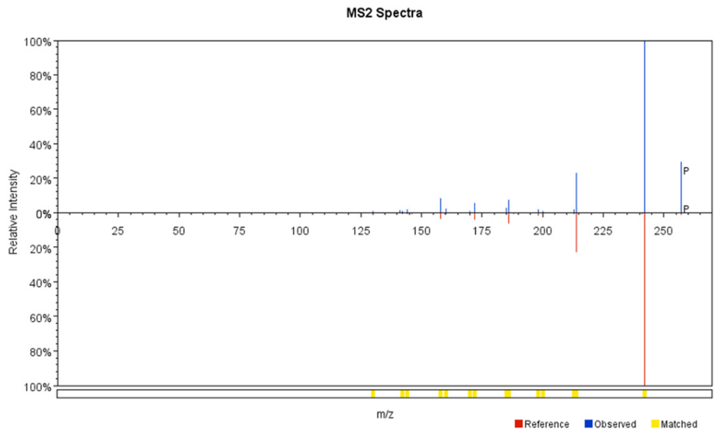

(ah)

Figure S1. Representative MS/MS spectral mirror plots of the tentatively identified compounds in HPE listed in Table 1. The upper blue spectra indicate the experimentally observed MS/MS spectra, whereas the lower red spectra indicate the corresponding reference spectra. The spectra support the tentative LC–MS/MS annotations of the following compounds: protocatechuic acid (**a**), neochlorogenic acid (**b**), protocatechuic aldehyde (**c**), procyanidin B1 (**d**), 5-*O*-coumaroylquinic acid (**e**), procyanidin B3 (**f**), catechin (**g**), 1-*O*-feruloylglucose (**h**), chlorogenic acid (**i**), cryptochlorogenic acid (**j**), epicatechin (**k**), 3-*O*-coumaroylquinic acid (**l**), 4-*O*-coumaroylquinic acid (**m**), mangiferin (**n**), *p*-coumaric acid (**o**), 1-*O*-coumaroylquinic acid (**p**), myricetin-3-*O*-galactoside (**q**), myricetin-3-*O*-glucoside (**r**), rutin (**s**), quercetin 3-*O*-glucuronide (**t**), hyperoside (**u**), cynaroside (**v**), nicotiflorin (**w**), avicularin (**x**), spiraeoside (**y**), quercitrin (**z**), 4,5-di-*O*-caffeoylquinic acid (**aa**), quercetin-3-*O*-glucose-6″-acetate (**ab**), quercetin (**ac**), luteolin (**ad**), kaempferol (**ae**), amentoflavone (**af**), 3-hydroxy-7,2′,4′-trimethoxyflavone (**ag**), and isogentisin (**ah**).

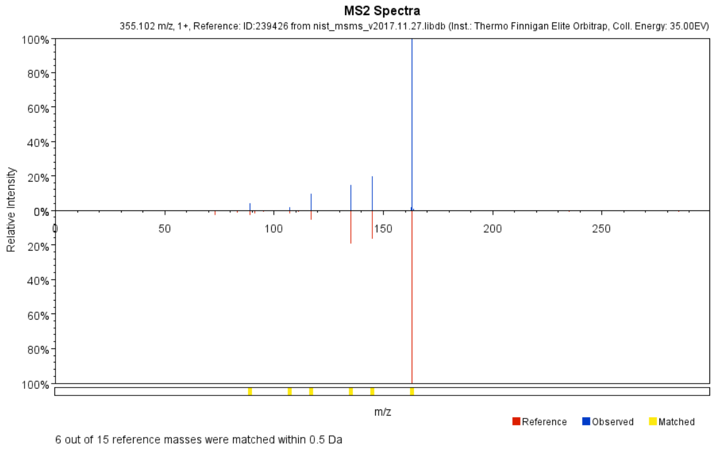
MS2 Spectra
355.102 m/z, 1+, Reference: ID:239426 from nist_msms_v2017.11.27.libdb (Inst.: Thermo Finnigan Elite Orbitrap, Coll. Energy: 35.00EV)
Relative Intensity
m/z
Reference
Observed
Matched
6 out of 15 reference masses were matched within 0.5 Da

(a)

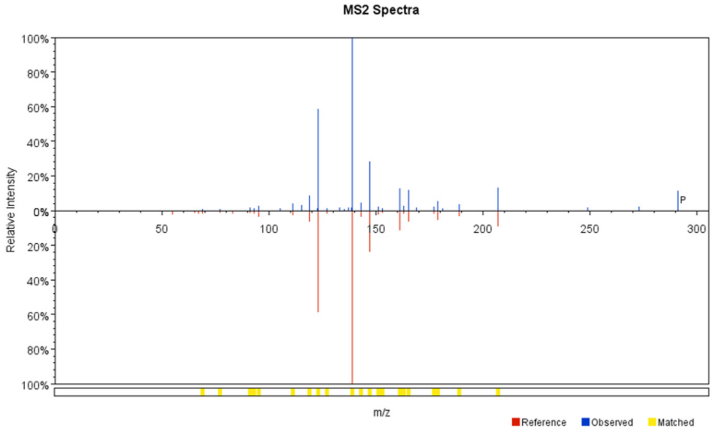
MS2 Spectra
Relative Intensity
m/z
P
Reference
Observed
Matched

(b)

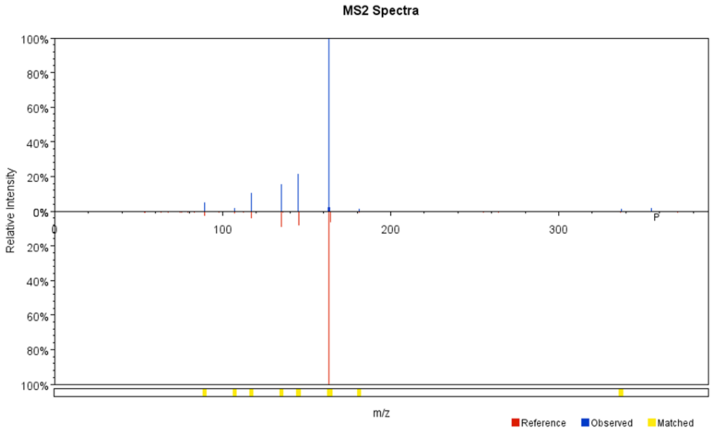
MS2 Spectra
Relative Intensity
m/z
P
Reference
Observed
Matched

(c)

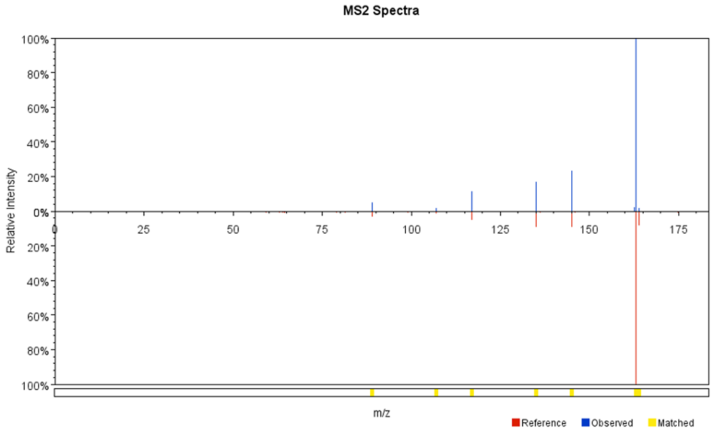
MS2 Spectra
Relative Intensity
m/z
Reference
Observed
Matched

(d)

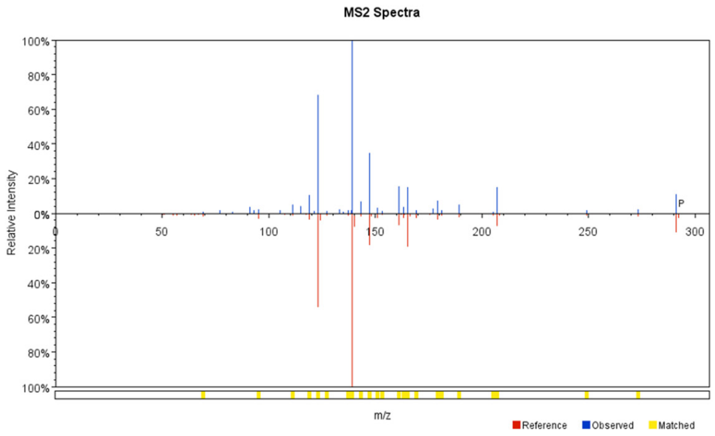
MS2 Spectra
Relative Intensity
m/z
Reference
Observed
Matched

(e)

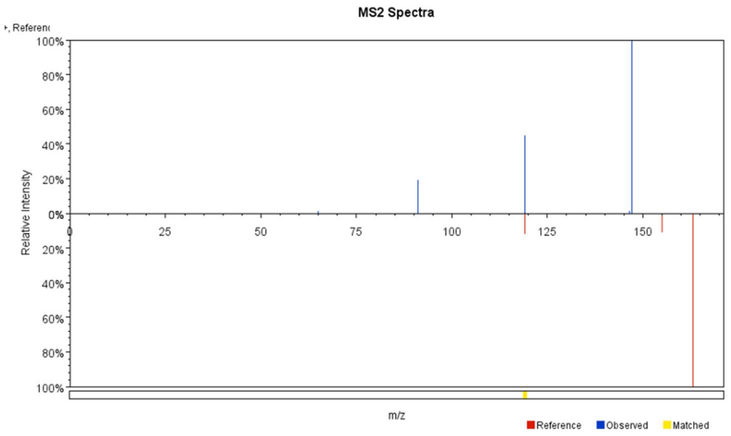
MS2 Spectra
Relative Intensity
m/z
Reference
Observed
Matched

(f)

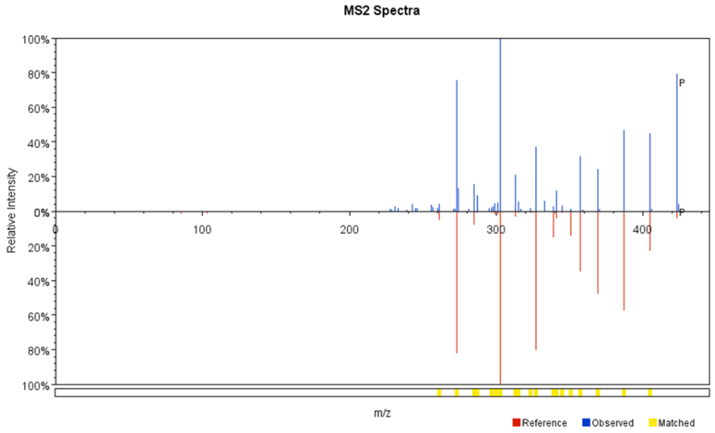
MS2 Spectra
Relative Intensity
m/z
Reference
Observed
Matched

(g)

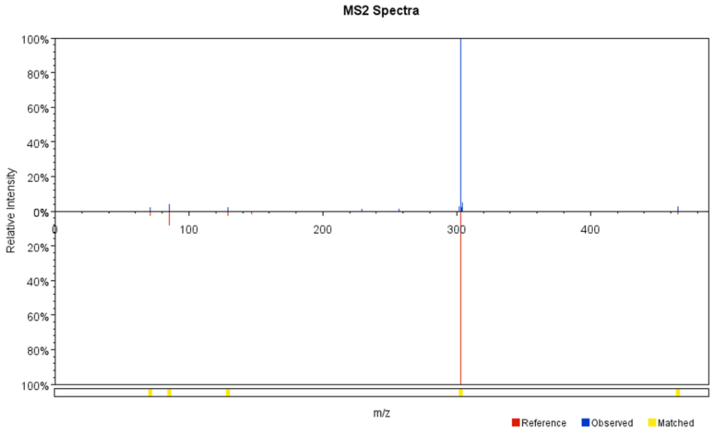
MS2 Spectra
Relative Intensity
m/z
Reference
Observed
Matched

(h)

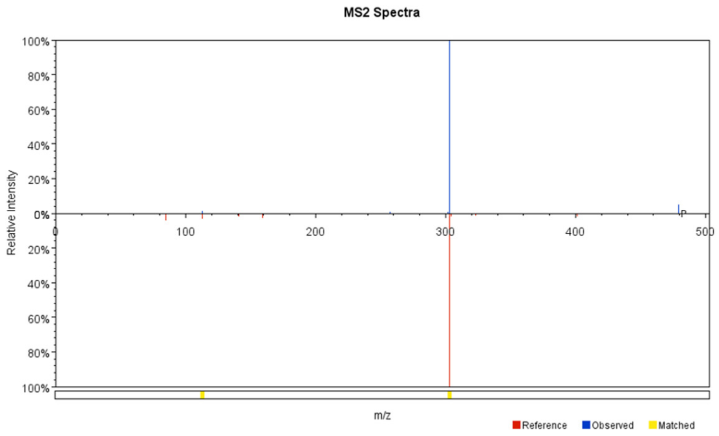
MS2 Spectra
Relative Intensity
m/z
Reference
Observed
Matched

(i)

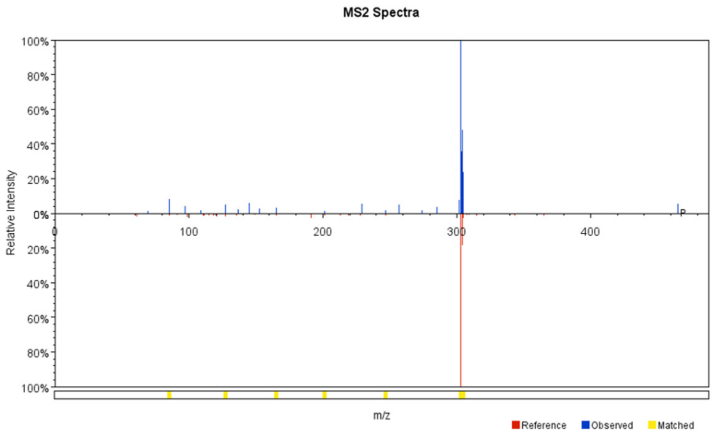
MS2 Spectra
Relative Intensity
m/z
Reference
Observed
Matched

(j)

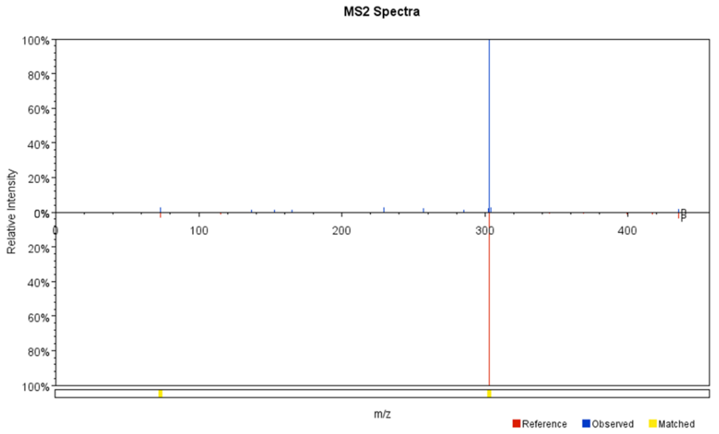
MS2 Spectra
Relative Intensity
m/z
Reference
Observed
Matched

(k)

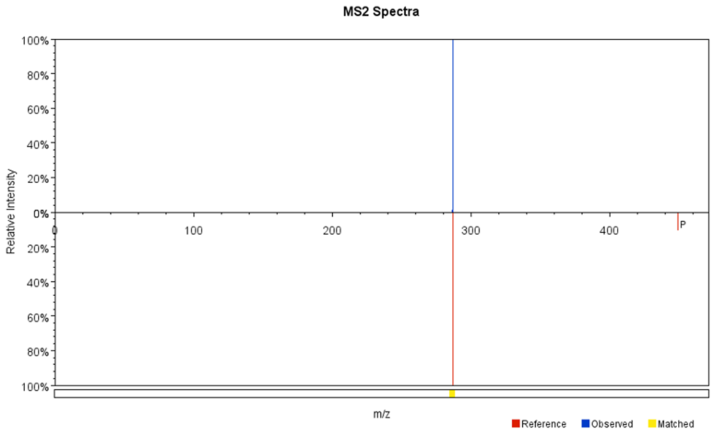
MS2 Spectra
Relative Intensity
m/z
Reference
Observed
Matched

(l)

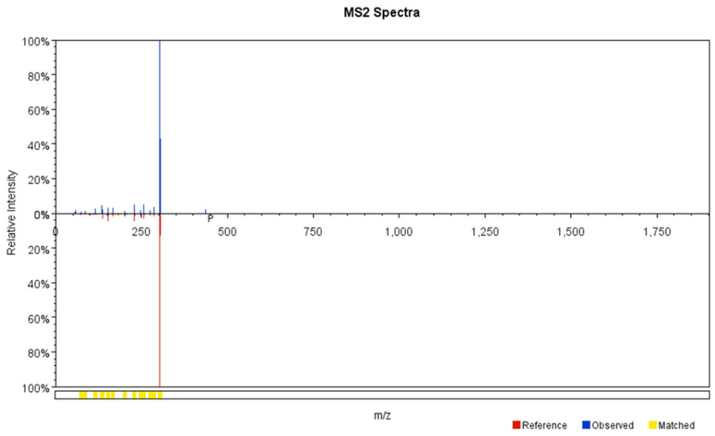
MS2 Spectra
Relative Intensity
m/z
Reference
Observed
Matched

(m)

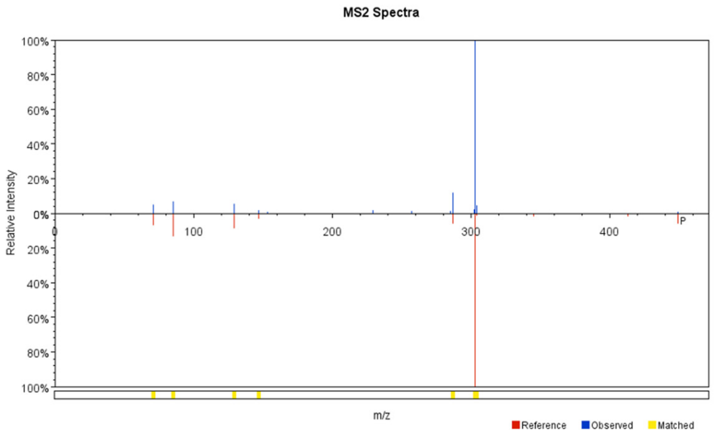
MS2 Spectra
Relative Intensity
m/z
Reference
Observed
Matched

(n)

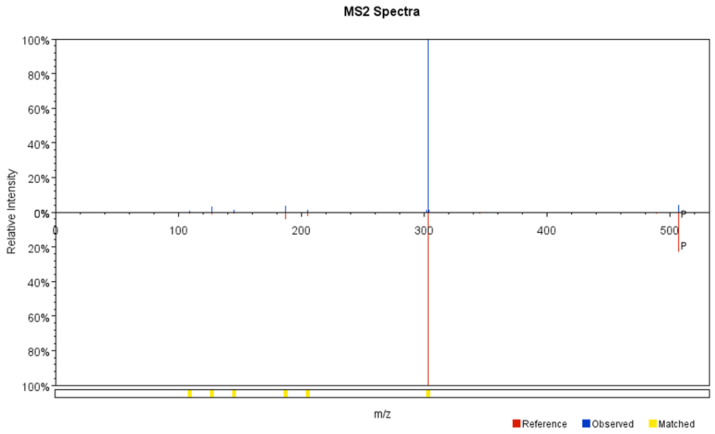
MS2 Spectra
Relative Intensity
m/z
Reference
Observed
Matched

(o)

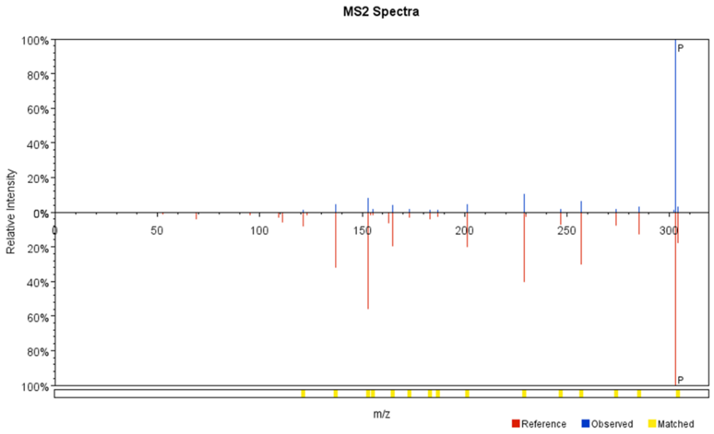
MS2 Spectra
Relative Intensity
m/z
Reference
Observed
Matched

(p)

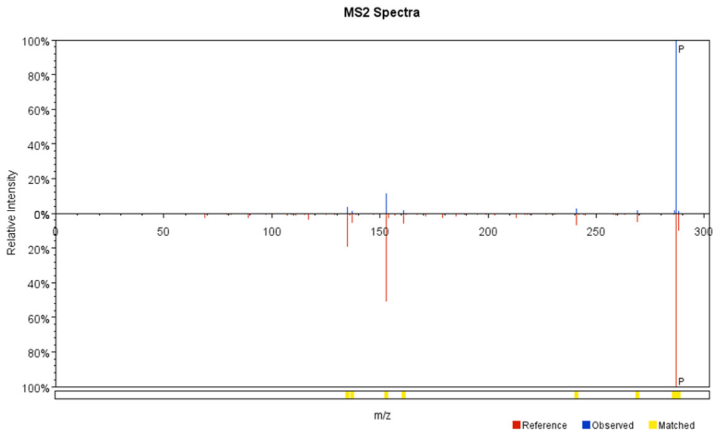

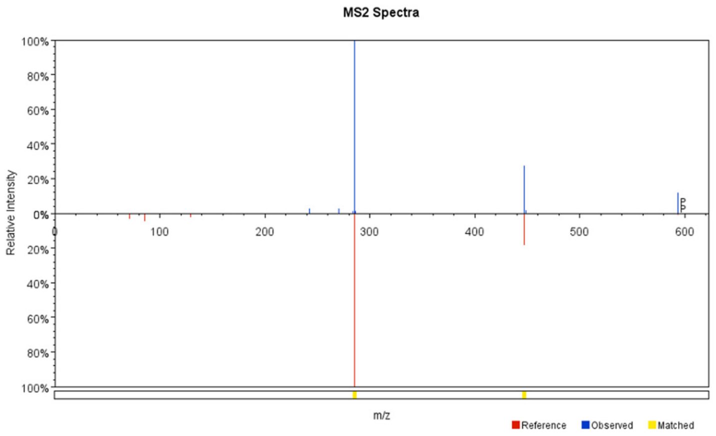

(**q**) (**r**)

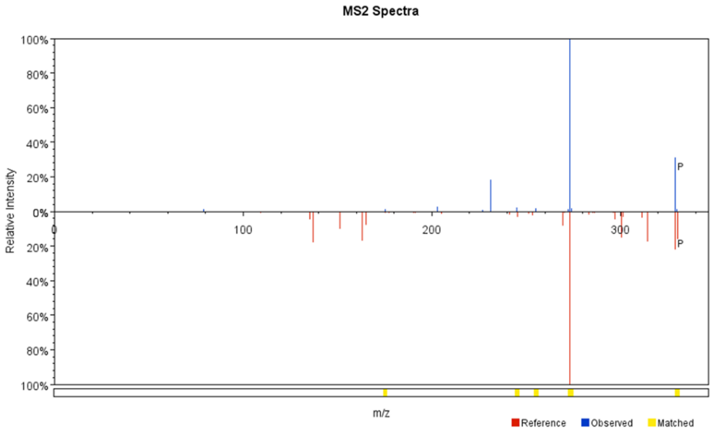

(**s**)

Figure S2. Representative MS/MS spectral mirror plots of the tentatively identified compounds in HPE listed in Table 2. The upper blue spectra indicate the experimentally observed MS/MS spectra, whereas the lower red spectra indicate the corresponding reference spectra. The spectra support the tentative LC–MS/MS annotations of the following compounds: neochlorogenic acid (**a**), catechin (**b**),

chlorogenic acid (**c**), cryptochlorogenic acid (**d**), epicatechin (**e**), 3-*O*-coumaroylquinic acid (**f**), mangiferin (**g**), rutin (**h**), quercetin 3-*O*-glucuronide (**i**), hyperoside (**j**), guaiaverin (**k**), cynaroside (**l**), avicularin (**m**), quercitrin (**n**), quercetin-3-*O*-glucose-6″-acetate (**o**), quercetin (**p**), luteolin (**q**), linarin (**r**), and 3-hydroxy-7,2′,4′-trimethoxyflavone (**s**).
